# Oxidation of metallic Cu by supercritical CO_2_ and control synthesis of amorphous nano-metal catalysts for CO_2_ electroreduction

**DOI:** 10.1038/s41467-023-36721-8

**Published:** 2023-02-25

**Authors:** Chunjun Chen, Xupeng Yan, Yahui Wu, Xiudong Zhang, Shoujie Liu, Fanyu Zhang, Xiaofu Sun, Qinggong Zhu, Lirong Zheng, Jing Zhang, Xueqing Xing, Zhonghua Wu, Buxing Han

**Affiliations:** 1 Beijing National Laboratory for Molecular Sciences, Key Laboratory of Colloid and Interface and Thermodynamics, Institute of Chemistry, Chinese Academy of Sciences, 100190 Beijing, China; 2 School of Chemistry and Chemical Engineering, University of Chinese Academy of Sciences, 100049 Beijing, China; 3 Chemistry and Chemical Engineering of Guangdong Laboratory, 515063 Shantou, China; 4 Institute of High Energy Physics, Chinese Academy of Sciences, 100049 Beijing, China; 5 Physical Science Laboratory, Huairou National Comprehensive Science Center, No. 5 Yanqi East Second Street, 101400 Beijing, China; 6 Shanghai Key Laboratory of Green Chemistry and Chemical Processes, School of Chemistry and Molecular Engineering, East China Normal University, 200062 Shanghai, China

**Keywords:** Green chemistry, Electrocatalysis, Electrocatalysis

## Abstract

Amorphous nano-metal catalysts often exhibit appealing catalytic properties, because the intrinsic linear scaling relationship can be broken. However, accurate control synthesis of amorphous nano-metal catalysts with desired size and morphology is a challenge. In this work, we discover that Cu(0) could be oxidized to amorphous Cu_x_O species by supercritical CO_2_. The formation process of the amorphous Cu_x_O is elucidated with the aid of machine learning. Based on this finding, a method to prepare Cu nanoparticles with an amorphous shell is proposed by supercritical CO_2_ treatment followed by electroreduction. The unique feature of this method is that the size of the particles with amorphous shell can be easily controlled because their size depends on that of the original crystal Cu nanoparticles. Moreover, the thickness of the amorphous shell can be easily controlled by CO_2_ pressure and/or treatment time. The obtained amorphous Cu shell exhibits high selectivity for C2+ products with the Faradaic efficiency of 84% and current density of 320 mA cm^−2^. Especially, the FE of C2+ oxygenates can reach up to 65.3 %, which is different obviously from the crystalline Cu catalysts.

## Abstract (web summary)

Amorphous metal catalysts often exhibit appealing catalytic properties, however, accurate control synthesis of amorphous nano-metal catalysts is a challenge. Here, the authors report a feasible strategy to prepare the amorphous Cu catalysts by supercritical CO_2_ treatment followed by electroreduction. The resulted catalyst shows high selectivity towards multi-carbon products for CO_2_ electroreduction.

## Introduction

The amorphous (long-range disordered structure) nano-metal catalysts are very attractive because they often show appealing physical and catalytic properties^1–3^. Compared to crystalline materials, the amorphous structure exhibits contrasting atomic arrangement and abundant low-coordinated atoms, which can decrease the energy barriers for the reaction and enhance the adsorption of intermediates, and the intrinsic linear scaling relationship can be broken^4–6^. However, the conventional preparation methods for amorphous metal, such as composite explosive welding, mechanical alloying, and arc-melting pure metals, often involved high temperature, leading to large particle size and low defects, which significantly decrease the active sites for reaction^7^. In recent years, some amorphous metal catalysts have been prepared by one-step synthesis, which involves too fast or too slow reaction rate^7,8^. However, the size of the amorphous metal catalyst is hard to be controlled using the one-step method, because the nucleation and growth rate cannot be managed. Thus developing controlled new preparation methods and exploring the formation mechanism of amorphous metals are of great importance.

In recent years, supercritical (SC) CO_2_ has been used to control the formation of special materials, because the properties of the materials can be well adjusted by tuning CO_2_ pressure, and the CO_2_ can be easily removed by depressurization^9,10^. For example, the thin graphene layers can be stripped from the bulk materials using SC CO_2_ strategy, because the CO_2_ can insert into the interlayers of the bulk two-dimensional materials^11–13^. In particular, the amorphous MoO_3_, WO_3_@x and VS_2_ nanosheets have been fabricated using SC CO_2_^14–16^.

In this work, amorphous Cu is prepared by a combination of SC CO_2_ treatment of Cu nanoparticles (Cu-np) followed by electroreduction. The formation process of the amorphous Cu is simulated by using machine learning (ML). We find that the Cu can be oxidized to amorphous Cu_x_O species by SC CO_2_, and the formation rate of Cu_x_O can be easily controlled by pressure. Then the amorphous Cu is produced by electroreduction. The obtained amorphous Cu exhibits high selectivity for C2+ oxygenates rather than the ethylene from electroreduction CO_2_. The Faradaic efficiency (FE) of C2+ oxygenates can reach up to 65.3% over the amorphous Cu with a current density of 320 mA cm^−2^. From the in situ surface-enhanced Raman spectroscopy (SERS) and density functional theory (DFT) calculations, we find that the obtained amorphous Cu exhibits high activity for electroreduction CO_2_ to CO and high surface coverage of *CO intermediates, which can enhance the selectivity for C2+ oxygenates. In addition, the Co nanosheet with an amorphous shell is also prepared by SC CO_2_, suggesting the generality of our strategy.

## Results

### Synthesis and characterization of Cu nanoparticles and amorphous Cu

**Fig. 1:**
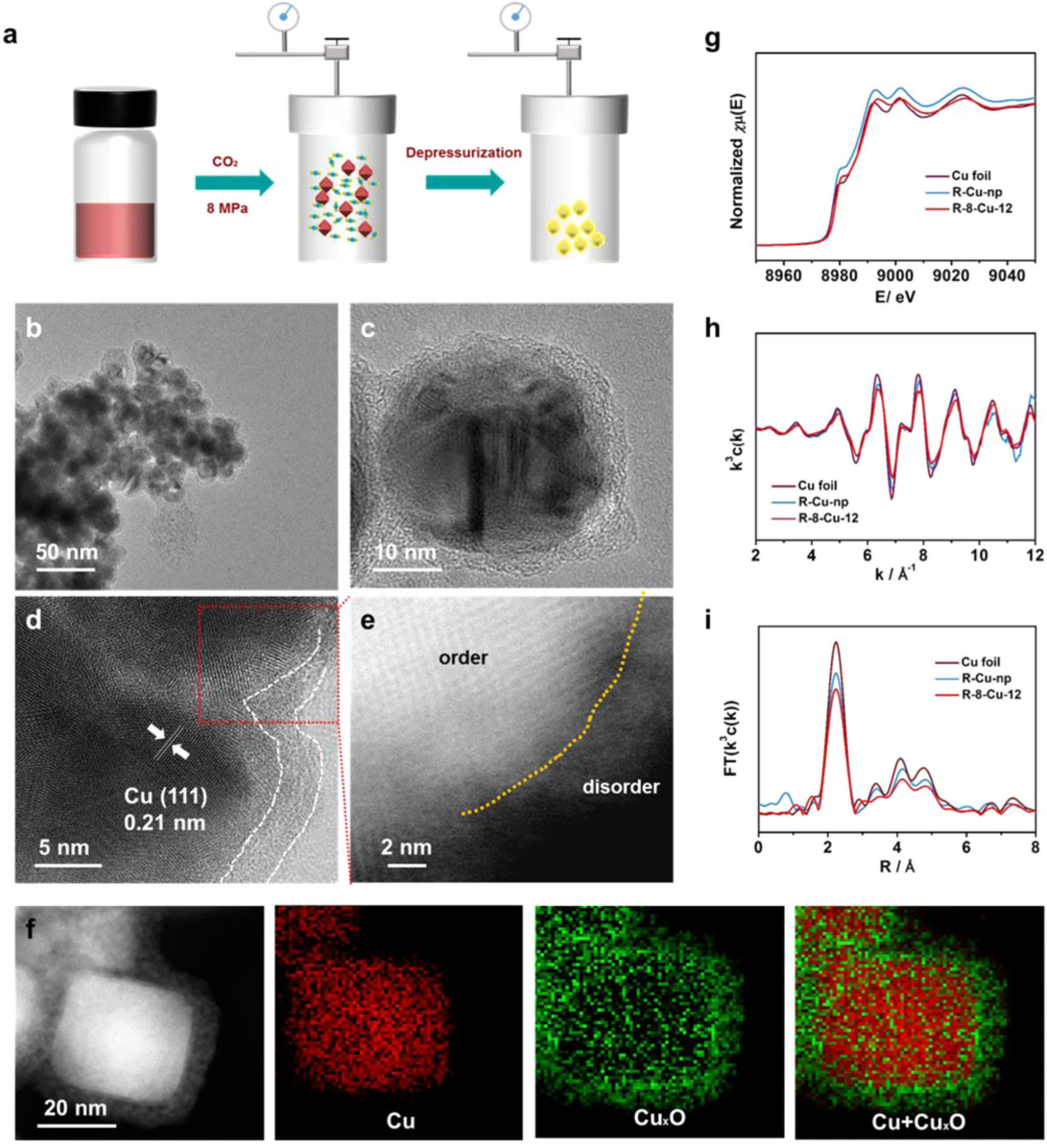
Structural characterization of the catalysts. **a** The schematic of the preparation of amorphous catalysts. **b**, **c** The TEM images of 8-Cu-12. **d** HR-TEM images of the 8-Cu-12. **e** HAADF-STEM images of the 8-Cu-12. **f** EELS maps of Cu and Cu_x_O and their overlay in 8-Cu-12. **g** The XANES spectra at the Cu K-edge for different catalysts. **h** Cu K-edge extended XAFS oscillation function **k**^3^c(**k**). **i** Fourier-transformed Cu K-edge EXAFS spectra for different catalysts.

Firstly, the Cu nanoparticles (Cu-np) were prepared using a method similar to that reported^17^. The obtained Cu-np had a size of about 40 nm (Supplementary Fig. S1), and they possessed Cu (111) facet and abundant twin boundaries on the surface, which was consistent with the previous reports^17^. For the synthesis of amorphous catalysts, the obtained Cu-np was first dispersed in methanol solution and transferred into an autoclave, then CO_2_ was charged into the autoclave until the desired pressure was reached, and the pressure was kept for desired time. The amorphous materials were obtained after depressurization (Fig. 1a). For clarity, x-Cu-y refers to the obtained Cu materials obtained at different pressure and reaction time, *x* represents the pressure of CO_2_ and *y* represents the reaction time. Interestingly, a core-shell structure was obtained after the reaction for 12 h (Fig. 1b, c) at 8 MPa (8-Cu-12). From the high-resolution TEM (HR-TEM) image, we can observe that the shell of 8-Cu-12 exhibited amorphous structure, and the thickness of the amorphous shell was about 4 nm (Fig. 1d). In contrast, the core exhibited crystal structure, and the Cu (111) can be observed. In addition, the amorphous shell structure was also verified by the Fast Fourier transform pattern (Supplementary Fig. S2). As characterized by high-angle annular dark-field scanning transmission electron microscopy (HAADF-STEM), the disordered arrangement of atoms was observed in the area of the amorphous shell (Fig. 1e), indicating that the amorphous structure was obtained by the processing of SC CO_2_. In addition, X-ray diffraction (XRD) patterns were used to study the crystal structure of the 8-Cu-12, and it can be observed that the peak of Cu (111) was weaker than that of the Cu-np (Supplementary Fig. S3). Moreover, we can observe that the size of the 8-Cu-12 was also about 40 nm, which is similar to that of Cu-np, indicating that the size of the particles with amorphous shell can be easily controlled because their size depends on that of the original crystal Cu nanoparticles.

Furthermore, the species of the amorphous shell in 8-Cu-12 were identified by the semi-in-situ X-ray photoelectron spectroscopy (XPS)^18^ and X-ray absorption spectroscopy (XAS). In order to minimize the interference from air oxidation, the samples were carefully kept in a glove box. It can be known from the high-resolution Cu 2*p* spectrum and Cu LMN Auger electron spectrum (Supplementary Figs. S4, S5), the surface of the Cu-np was Cu^+^ and Cu^0^ species. However, the Cu^2+^ species were predominant on the surface of 8-Cu-12. In addition, the proportions of different species were calculated by linear fitting of the X-ray absorption near-edge structure (XANES) spectra (Supplementary Fig. S6). For the Cu-np, the metallic Cu was the main species, while CuO was comparable to that of metallic Cu in the 8-Cu-12. Because of the core of the 8-Cu-12 was the metallic Cu (Fig. 1d), we can assume that the amorphous shell of 8-Cu-12 was Cu_*x*_O species rather than the metallic Cu. Although the samples may be oxidized by air during testing or preparation, the 8-Cu-12 contained much more Cu_x_O species than that of the Cu-np. Furthermore, Cu valance state mapping based on electron energy loss spectroscopy (EELS) spectrum imaging shows that the core was Cu and the amorphous shell was Cu_*x*_O (Fig. 1f and Supplementary Fig. S7). Thus we can conclude that the metallic Cu can be oxidized to amorphous Cu_x_O species by the SC CO_2_. Furthermore, the Cu-np was oxidized by the O_2_ at 8 MPa, we can observe that some crystal CuO appeared on the surface of Cu-np, and no amorphous species appeared (Supplementary Fig. S8). Thus we can assume that the SC CO_2_ has unique oxidation for the Cu.

Then the Cu-np and 8-Cu-12 were reduced by the cyclic voltammetry method. From the XANES and k^3^-weighted Fourier-transformed (FT) extended X-ray absorption fine structure (EXAFS) spectra (Fig. 1g, i), we can know that both the reduced Cu-np (R-Cu-np) and reduced 8-Cu-12 (R-8-Cu-12) exhibited similar spectrum features to Cu foil, and the Cu–O peaks disappeared, indicating that the Cu_*x*_O species can be fully reduced to metallic Cu after the electrochemical reduction. Compared to the R-Cu-np, R-8-Cu-12 showed a decreased intensity in k-space (Fig. 1h), indicating its lower coordination number (CN) and relatively higher disorder^19^. From EXAFS spectra, the intensity of Cu–Cu bond over R-8-Cu-12 was lower than that over R-Cu-np, demonstrating that the R-8-Cu-12 exhibited a lower coordination number than that of R-Cu-np. By fitting for EXAFS (Supplementary Fig. S9, and Table S1), the coordination number of Cu–Cu is 8.6 for R-8-Cu-12, which is less than that of R-Cu-np (9.3). The reason may be that the R-8-Cu-12 exhibits the amorphous Cu shell. In addition, the R-8-Cu-12 was characterized by the HR-TEM and HAADF-STEM, which showed that the amorphous shell existed (Supplementary Fig. S10). Thus, we can know that the amorphous Cu shell was prepared by using the SC CO_2_ and electroreduction.

Furthermore, the crystal facets on the surface of R-Cu-np and R-8-Cu-12 were probed by electroadsorption of hydroxide (OH_ads_)^20,21^. For the R-Cu-np, the (100), (110), and (111) OH_ads_ peaks were observed, and the intensity of (111) OH_ads_ feature was higher than that of (100) and (110), which suggests a high surface density of (111) on R-Cu-np (Supplementary Fig. S11). In contrast, for the R-8-Cu-12, the (111) and (110) OH_ads_ peaks disappeared, and the (100) OH_ads_ peak was weak, showing the amorphous Cu nature.

### Evolution of amorphous Cu_x_O shell with reaction time and CO_2_ pressure

In order to investigate the formation of amorphous Cu_*x*_O shell, different control experiments were conducted. First, the formation of the amorphous shell was investigated by varying pressure. It is known from the HR-TEM images that no amorphous shell was observed when the pressures of CO_2_ were 4 MPa (4-Cu-12) and 6 MPa (6-Cu-12), indicating that the SC pressure (7.38 MPa) is necessary for the formation of amorphous structure (Supplementary Figs. S12, S13). The main reason is that the density of CO_2_ is much higher above critical pressure than that at lower pressure. In addition, the evolution of the amorphous shell was studied at different reaction time at 8 MPa. We can observe that the amporphous Cu shell formed when the reaction time was 4 h (8-Cu-4), and the thickness of the amorphous shell was 2 nm (Supplementary Fig. S14). When the reaction time extended to 16 h (8-Cu-16), the thickness of the amorphous shell was about 4 nm (Supplementary Fig. S15), which is similar to that at 12 h. From the above result, we can get a conclusion that the amorphous shell can be produced by SC CO_2_ treatment, and the thickness of the amorphous shell increased with the reaction time. However, when the reaction time exceeded 12 h, the thickness of the amorphous shell increased very slowly.

### Simulation of the formation of amorphous Cu

A study of the mechanisms for the amorphization usually calls for ab initio molecular dynamics (AIMD). However, AIMD is prohibitively expensive. A compromise is the introduction of a neural network (NN). It represents functions of many variables in a continuous way and interpolates within the training set, which allows us to obtain a faithful representation of the ab initio potential energies and forces, at a much reduced cost. Practically, NNs based molecular dynamics (MD) is an “equivalently accurate, but faster” AIMD. Till today, the structure of NN used to represent DFT result has gradually reached a paradigm, which is the high-dimensional neutral network potentials (HDNNPs) proposed by Behler and Parrinello^22–24^. It decouples the total energy of the system to a sum of atomic energies. And using the concept of “nearsightedness” to regard the atomic potential as the functional of the local chemical environment up to a cutoff radius and are computed by individual atomic neural networks. Thus, the HDNNPs based MD were used to understand the amorphization of Cu under SC CO_2_ in this work. The associated details are provided in Supplementary S1-S2 of Computational Method. Briefly, the simulation was carried out inside a rigid cubic box with the edge length of 8.8 nm. The Cu with a regular octahedron (length of 4.2 nm) was put inside this box, which is comprised of the Cu (111) and the step of Cu(111)×Cu(111). Then 3000 CO_2_ molecules were put inside the cubic box, and the density of CO_2_ was 0.316 g cm^−3^, which was matched well with the density of SC CO_2_. During the simulation, the system was first equilibrated under canonical ensemble condition (NVT) imposed by a Nosé thermostat with a target temperature of 300 K for 20 ps, and a high temperature was used to overcome the too slow amorphization process in experiments (forming 2 nm of amorphous Cu in 4 h). The temperatures were selected based on a temperature test (Supplementary Figs. S16, S3 of Computational Method) that shows within such temperatures. Then the obtained 9000 DFT data from different models and temperatures were used to train the neutral network (NN) for simulating the formation of amorphous Cu.

As for the NN, we adopt that from the Deep Potential-Smooth Edition (DeepPot-SE). It is similar to the traditional BPNN. But making some improvement in the expression of the atomic local environment. In DeepPot-SE, the atomic local environment is first constructed by a local coordinate frame to keep the translational, rotational, and permutational symmetries of the environment^25^. This local coordinate frame is then washed up by a local embedding network to get a smooth edition^26^. Such a description allows the potential function to maintain continuity and derivability while ensuring the rotation-translation symmetry. And compared to similar HDNNPs such as DTNN and SOAP^27^, it can reduce the artificial input parameters while ensuring the same learning accuracy. The associated loss function is

1 $$L\left({p}_{\varepsilon },{p}_{f}\right)={p}_{\varepsilon }\Delta {\epsilon }^{2}+\frac{{p}_{f}}{3N}\mathop{\sum}\limits_{i}{\left|\triangle {F}_{i}\right|}^{2}$$

Δ denotes the difference between the NN prediction and the training data, *N* is the number of atoms, ϵ is the atomic energy, *F*_*i*_ is the force exerted on atom *i*. *p*_*ϵ*_ and *p*_*f*_, are adjustable prefactors. In order to minimize the loss function in a well-balanced way, we progressively increase *p*_*ϵ*_ and decrease *p*_*f*_, so that the force term dominates at the beginning, while energy terms become important at the end.

The generation process was recorded in Supplementary, *movie 1, *movie 2 and Fig. S17. We can find that the formation of the amorphous Cu can be roughly divided into three steps: the surface oxidation, O atomic penetration, and O departing.

**Fig. 2:**
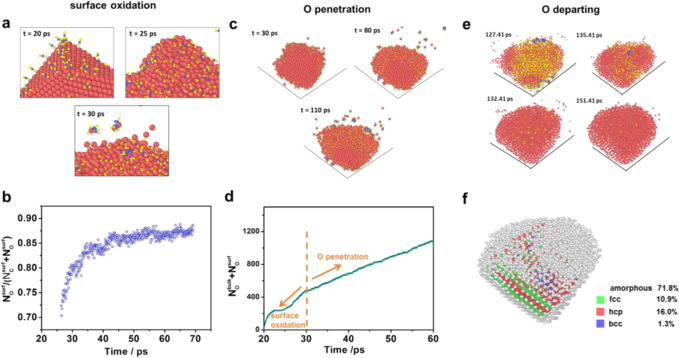
The simulated mechanism for the amorphization. **a** The snapshots of the surface oxidation process. **b** The fraction of surface O in the total number of surface C and O. **c** The snapshots of the slices of O atoms penetration process. **d** The time-dependent total number of the O that bind with Cu, including the O on the surface and bulk. **e** The snapshots for the O departing process. **f** The results of the polyhedral template matching of the final structure. It is plotted by OVITO. For all the pictures, the red, yellow, and blue circle stand for Cu, O, and C, respectively.

For the first step, the surface oxidation means that the surface of Cu can be covered by the *O from the CO_2_. As shown in Fig. 2a and Supplementary, *movie 3, the Cu surface can be occupied by molecular CO_2_, and the CO_2_ dissociated to yield *CO and *O on the surface. Then the *O stayed on the surface of Cu, and the *CO gradually depart from the surface site. This is because that the *O adsorption is stronger than that of the *CO. Finally, the surface is almost covered completely by *O (Fig. 2b). In addition, there are some issues needed to be discussed. According to DFT results, the dissociation of a single CO_2_ molecule is quite exothermic (0.35 eV in energy from a *CO_2_ dissociation into *O and *CO). But the density of SC CO_2_ molecules around Cu surface is much larger (about 300 times) than that of gaseous CO_2_. It can largely increase the chance for the formation of *CO and *O.

For the second step, the *O gradually penetrated inside into the Cu lattice when the *O on the surface of Cu was overloaded (Fig. 2c and Supplementary, *movie 4). This is because the excess O species need new sites to locate, which drives the O penetration inside into the Cu lattice. Interestingly, the original fcc crystal structure disappeared with the O atomic penetration (Supplementary Fig. S18). As time goes on, the diffusion finally reached a steady state, as indicated by the fixed slope of Fig. 2d. These results show that the amorphous Cu_x_O shell can be obtained by the SC CO_2_ treatment which was consistent with the experiment results.

For the third step, the O departing means that the O was removed from the amorphous Cu_x_O, which was used to simulate the electrochemical reduction process in the experiments. During the electroreduction, the Cu_x_O can be quickly reduced. Thus the O atoms outside the Cu surface are artificially deleted. As shown in Fig. 2e, and Supplementary, *movie 5, the Cu atoms are disordered after the O departing, indicating that the amorphous Cu is obtained (Fig. 2f).

### The feasibility of this strategy

From the above results, we can deduce that the main reason for the formation of amorphous Cu_x_O is that the *CO_2_ can be broken into *CO and *O, and the *O can penetrate inside into the Cu lattice under SC CO_2_. This may be related to the reducibility and strong *O adsorption of Cu. Thus we can assume that the amorphous nano-metal catalysts with similar properties to copper can also be prepared by using the SC CO_2_. To confirm this hypothesis, the crystalline Co nanosheet and Ag nanoparticles were prepared according to the previous report^28,29^. The Co possessed similar chemical properties to Cu, as contrast, the Ag is more difficult to be oxidized than Cu, and it cannot be oxidized by SC CO_2_. After the treatment of SC CO_2_, an amorphous shell is observed over the Co nanosheet (Supplementary Fig. S19). However, no obvious changes were observed for Ag nanoparticles (Supplementary Fig. S20). This result proves that this strategy exhibits certain feasibility.

### Electrocatalytic performance of CO_2_ reduction

**Fig. 3:**
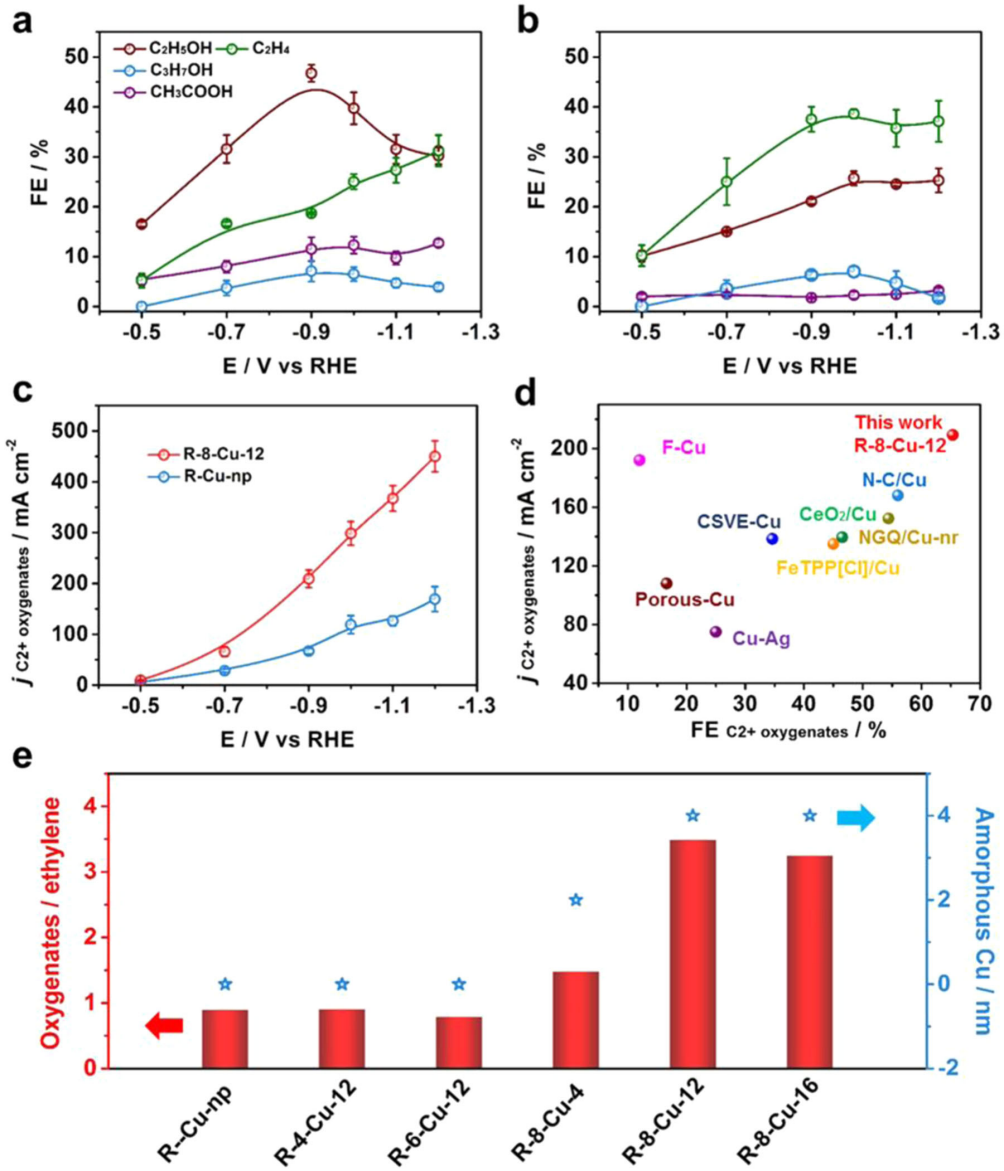
CO_2_RR performance for R-8-Cu-12 and R-Cu-np. **a** The distribution of C2 + products at different potentials over R-8-Cu-12. **b** The distribution of C2+ products at different potentials over R-Cu-np. **c** The partial current density of C2+ oxygenates at different potentials for R-8-Cu-12 and R-Cu-np. **d** Plot of C2+ oxygenates partial current density versus maximum C2+ oxygenates FE for various catalysts, and the source of literature are listed in supporting information (Table S2). **e** The relationship between the ratios of C2+ oxygenates/ethylene and the thickness of amorphous Cu shell. Error bars correspond to the standard deviation of three independent measurements.

According to the previous report, the Cu-based nanocatalysts are considered as the most promising electrocatalysts for producing C2+ products from electroreduction of CO_2_^30–33^. However, the selectivity for C2+ oxygenates is still ungratified over the Cu-based catalysts. This is because that the generation of C2+ oxygenates is unfavorable over the most common Cu (100) and Cu (111) facet. According to a previous report, the Cu (100) surface exhibited high selectivity toward ethylene, whereas the Cu (111) surface has been shown to favor methane formation^30^. In recent years, the selectivity of C2+ products was enhanced from CO_2_RR by the oxide derived copper^34–37^. However, the oxide-derived Cu is still a crystal Cu catalyst, and the major C2+ product was the C_2_H_4_. Thus, we can assume that the amorphous Cu will exhibit a special selectivity. The electrocatalytic performance of the catalysts was evaluated in a flow cell by the controlled potential electrolysis experiments, as reported in our previous work^38^. We can find that the R-8-Cu-12 exhibited a high selectivity for C2+ products, the FE of C2+ products (FE_C2+_) could reach up to 84% with a current density of 320 mA cm^−2^ at −0.9 V vs RHE (Supplementary Fig. S21). In particular, the FE of C2+ oxygenates could reach 65.3% (ethanol 46.7%, *n*-propanol 7.1% and acetate 11.5 %), which is about 3 times larger than that of ethylene (Fig. 3a). In contrast, for the R-Cu-np, the FE of ethylene (37.5%) was higher than that of C2+ oxygenates (35.1%) at −0.9 V vs. RHE (Fig. 3b). In addition, the partial current density of C2+ oxygenates over R-8-Cu-12 could reach 209.2 mA cm^−2^ at −0.9 V vs. RHE, which is about 3 times of that over R-Cu-np (Fig. 3c). The distribution of all products is listed in Supplementary Fig. S22, we can observe that the hydrogen evolution reaction (HER) was suppressed over the R-8-Cu-12, indicating more CO_2_ can be reduced. In addition, we can observe that the FE of CO over R-8-Cu-12 was lower than that over R-Cu-np, and the FE of C2+ products over R-8-Cu-12 was higher than that over R-Cu-np. Thus, we can conclude that more CO was used for dimerization over the R-8-Cu-12. Compared with the state-of-the-art catalysts, the FE and partial current density for C2+ oxygenates over R-8-Cu-12 is one of the highest (Fig.3d and Supplementary Table S2). These results indicate that the activity and selectivity of C2+ oxygenates can be significantly enhanced over the amorphous Cu.

In addition, the electrocatalytic performance of CO_2_ reduction over different x-Cu-y catalysts was also studied. The FE of ethylene was still the main C2+ product for R-4-Cu-12 and R-6-Cu-12. In contrast, the FE of C2+ oxygenates became the main products for R-8-Cu-4 and R-8-Cu-16 (Supplementary Fig. S23–S26). Combined with the results of HR-TEM, it can be assumed that the increase of selectivity for C2+ oxygenates was associated with the amorphous shell. As shown in Fig. 3e, the ratios of C2+ oxygenates and ethylene were dependent on the thickness of the amorphous shell, showing that the amorphous Cu shell played a key role in the production of C2+ oxygenates.

The isotopic electrolysis experiments were carried out using the isotope-labeled ^13^CO_2_ to verify the source of the products. It can be known from ^1^H NMR spectra that the H signal of the products split into two group peaks due to the coupling effect of H-^13^C atom (Supplementary Fig. S27)^39^, indicating that the products were derived from the CO_2_.

Furthermore, the stability of R-8-Cu-12 was investigated using the polytetrafluoroethylene (PTFE) as a gas diffusion layer (GDL), due to the carbon-based gas diffusion electrode will become flooded as its hydrophobicity was lost during operation^40^. The FE of C2+ oxygenates over R-8-Cu-12 was about 60% under PTFE, which is also higher than that of ethylene (Supplementary Fig. S28). There was no obvious change in both current density and FE of the products at −0.9 V vs. RHE for 50 h, indicating that the R-8-Cu-12 exhibited exellent stability. In addition, we can observe that the change of the surface of amorphous Cu catalyst was not notable, suggesting that the catalyst was stable during CO_2_RR (Supplementary Fig. S29).

Previous reports indicate that the electrochemical active surface areas (ECSAs) play a crucial role in the selectivity of C2+ oxygenates products^41^. The ECSAs of the R-Cu-np and R-8-Cu-12 were estimated by measuring double-layer capacitance and Pb underpotential deposition (Pb_UPD_) method^28,42^. The ECSAs of R-8-Cu-12 were similar to that of the R-Cu-np (Supplementary Figs. S30, S31), indicating that the remarkable increase of C2+ oxygenates products over the R-8-Cu-12 resulted from its amorphous nature.

**Fig. 4:**
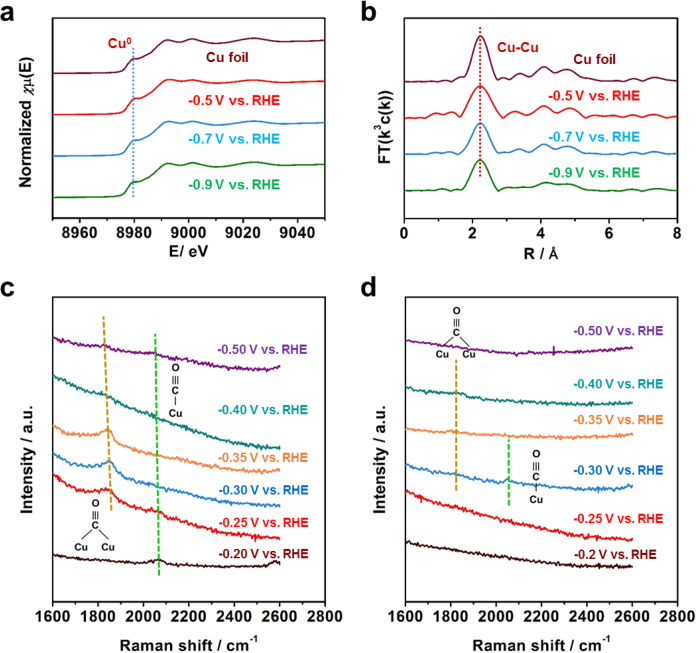
The operando and in situ SERS for R-8-Cu-12 and R-Cu-np. **a** The operando XANES spectra at the Cu K-edge for R-8-Cu-12 at different potentials during CO_2_ electrolysis. **b** The corresponding Fourier transforms FT(**k**^3^w(**k**)) for R-8-Cu-12 at different potentials during CO_2_ electrolysis. **c** The in situ SERS spectra over R-8-Cu-12 at different potentials during CO_2_ electrolysis. **d** The in situ SERS spectra over R-Cu-np at different potentials during CO_2_ electrolysis.

Due to the coordination environment of Cu plays a vital role on the activation of CO_2_ and the adsorption of intermediates^43^, operando XAS was used to monitor the local structure of the R-Cu-np and R-8-Cu-12 during CO_2_ electrolysis. For both R-Cu-np and R-8-Cu-12, only peaks corresponding to metallic Cu were observed at a negative potential (Fig. 4a, b and Supplementary Fig. S32, S33), indicating that the metallic Cu was the catalytic site in CO_2_ electroreduction. For comparison, the quantified Cu–Cu coordination numbers of the R-Cu-np and R-8-Cu-12 were fit using the ARTEMIS programs of IFEFFIT during CO_2_ electrolysis (Supplementary Fig. S34, S35 and Table S1). It was found that the R-8-Cu-12 exhibited lower Cu–Cu coordination number than that of R-Cu-np at each potential, and no obvious difference of Cu–Cu coordination number was observed for the R-8-Cu-12 at different potentials. Thus we can draw a conclusion that the R-8-Cu-12 possessed low-coordinate atoms during CO_2_ electrolysis, which is favorable to the activation of the CO_2_ and adsorption of intermediates.

Furthermore, in situ SERS was used to track the intermediates adsorption on the catalyst surface. Raman spectra were collected during the CO_2_ electrolysis at a series of applied potentials, as reported in our previous work^44^. The Raman peak located at 524 cm^−1^ appeared on both R-8-Cu-12 and R-Cu-np (Supplementary Fig. S36-S37), which corresponds to the adsorption of preliminary intermediates (such as *CO_2_ or *OCO^-^) on Cu^44,45^. For the R-8-Cu-12, the peak began to be observed at −0.2 V vs. RHE. However, the adsorption occurred at −0.25 V vs. RHE on R-Cu-np, indicating that CO_2_ can be activated over R-8-Cu-12 at lower potentials. In addition, for the R-8-Cu-12, the Raman peaks located at 1800-1850 cm^−1^ and 2024–2044 cm^−1^ began to be observed at −0.2 V vs. RHE or below, which correspond to the C ≡ O stretching mode of CO_bridge_ and CO_atop_ molecules on Cu, respectively (Fig. 4c)^46^. Interestingly, the bridge adsorption of CO was dominant, suggesting that the CO coverage was high on the amorphous Cu^47,48^, which can enhance the selectivity of oxygenates^49^. For the R-Cu-np, these peaks began to be observed at −0.3 V vs. RHE, and the intensity of these peaks was weak (Fig. 4d). These results demonstrated that the amorphous Cu structure exhibited high activity of converting CO_2_ to CO and more CO molecules were adsorbed on the surface of the catalyst. Meanwhile, the CO peak shifted to lower Raman wavenumbers with the increasing potential, which is caused by the electrochemical stark effect from the interaction between the applied electric field and adsorbed CO molecules^50^.

According to a previous report^51^, the selectivity and activity of C2+ oxygenates are related to the CO adsorption energy, energy barriers of dimerization and the coverage of CO on the surface of Cu. Thus, CO adsorption energies on the amorphous Cu model based on the amorphization simulation were studied. Due to the amorphous structure is not periodic, we used the cut models to count the CO adsorption energies, and the details are given in Supplementary S4 of Computational Method. 40 random positions were cut on the obtained amorphous Cu model (Supplementary Fig. S38 and Supplementary, *movie 6), and the CO adsorption energy distribution is shown in Supplementary Fig. S39. We can observe that the amorphous Cu possessed active sites that binds CO much stronger than Cu (111), which can suppress CO desorption and promote C2 + product generation. This may be because that the amorphous Cu possessed the active sites with low coordination number (Supplementary Fig. S40). Furthermore, the energy barriers of dimerization over the amorphous Cu were lower than that of the Cu (111), indicating the C-C coupling step can be enhanced over the amorphous Cu (Supplementary Fig. S41). In addition, CO coverage on the amorphous Cu (0.5) is significantly higher than that on the Cu (111) (Supplementary Fig. S42 and S5 of Computational Method), which may enhance the selectivity for C2+ oxygenates^49^.

## Discussion

In summary, the nanoparticles with amorphous Cu shell and crystal core were prepared by a combination of SC CO_2_ treatment and electroreduction. It can be known from the experiment and ML, the Cu is first oxidized to amorphous Cu_x_O species under SC CO_2_. Then the amorphous Cu was produced by electroreduction. The activity and selectivity of C2+ oxygenates can be improved significantly on the obtained amorphous Cu catalyst, and the FE of C2+ oxygenates was 65.3 % with the current density of 320 mA cm^−2^. According to the in situ SERS and DFT calculations, the amorphous Cu exhibited high activity for electroreduction CO_2_ to CO and high surface coverage of *CO intermediates, which are the main factors for the high efficiency to produce C2+ oxygenates. We believe that the oxidation of metals by SC CO_2_ the strategy to prepare amorphous metallic catalysts can also be used to prepare some other amorphous catalysts for efficient conversion of CO_2_.

## Methods

### Materials

Hexane, ethanol, methanol, HClO_4_ (70-72 wt%) dimethylformamide, AgNO_3_, and Ni foam were obtained from Sinopharm Chem. Reagent Co. Ltd. Copper(II) acetylacetonate (Cu(acac)_2_, 99.9%), Cobalt acetylacetonate (Co(acac)_3_, 99.9%), ethylene glycol, n-butylamine, L-Ascorbic acid (99%), Oleylamine (OAm) (80%), KOH (98%), PVP, sodium 2, 2-dimethyl-2-silapentane-5-sulfonate (DSS, 99%), Pb(ClO_4_)_2_ and phenol were purchased from Alfa Aesar China Co., Ltd. D_2_O (99.8% D) was purchased from Innochem Co., Ltd. N_2_ (99.999%) and CO_2_ (99.999%) were provided by Beijing Analytical Instrument Company. Deionized water was used in the experiments.

### Preparation of Cu-np

Typically, Copper acetylacetonate (44 mg) and l-ascorbic acid (210 mg) were added into Oleylamine (20 mL), and the mixture was sonicated for 60 min. The mixture was transferred to a metal bath under 130 °C for 4 h, and then cooled to room temperature. The obtained colloidal products were washed by hexane/ethanol solvents several times and dispersed in methanol solution (1 mL) for store.

### Preparation of Co nanosheet

Typically, Cobalt acetylacetonate (100 mg) was added into a solution of dimethylformamide (20 ml), H_2_O (4 ml) and *n*-butylamine (1 ml), and the mixture was sonicated for 30 min. Then, the mixture was transferred into a Teflon vessel held in a stainless steel autoclave. The autoclave was heated at 220 °C for 48 h, and then cooled to room temperature. The obtained colloidal products were washed by cyclohexane/ethanol solvents several times and dispersed in methanol solution (1 mL) for store. Before the reaction, the autoclave was pretreated by *n*-butylamine (20 ml) for 6 h and H_2_O (35 ml) for 12 h at 120 °C, respectively.

### Preparation of Ag nanoparticles

Typically, PVP (500 mg) was added into ethylene glycol (15 mL) under vigorous stirring. The mixture was heated up to180 °C for 30 min, and then cooled to and kept at 120 °C. 0.10 g of AgNO_3_ in ethylene glycol (5 mL) was added into the above mixture at 120 °C for 10 min under stirring. The mixture was cooled down to room temperature, then the acetone (100 ml) was added into the mixture. The obtained colloidal products were washed by H_2_O/ethanol solvents several times and dispersed in methanol solution (1 mL) for store.

### Preparation of amorphous Cu and Co

The obtained Cu-np methanol solution (1 mL) was transferred into a Teflon vessel held in a stainless steel autoclave of 15 mL. Then CO_2_ was charged into the autoclave under stirring to desired pressure at 35 °C for a suitable time, and the amorphous Cu_x_O catalysts were obtained after depressurization. Finally, the nanoparticles with amorphous Cu shell were prepared by the electrochemical reduction. The electrochemical reduction was performed by the cyclic voltammetry (CV) method, the potential range was from 0.0 V vs. RHE to −1.5 V vs. RHE, and the sweep rate was 100 mV s^−1^. Then amorphous Cu shell was obtained after 3 cycles of CV. Different catalysts were prepared by tuning the pressure and reaction time, the other conditions were the same with those above. In addition, the obtained Co nanosheet and Ag nanoparticle were treated by SC CO_2_.

### Characterization

The SEM and TEM images were carried out by the HITACHI S-4800 and JEOL JEM-2100F. X-ray powder diffraction (XRD) spectra were collected on a Rigaku D/max-2500 X-ray diffractometer. X-ray photoelectron spectroscopy (XPS) spectra were acquired on the Thermo Scientific ESCA Lab 250Xi. HAADF and EELS were recorded using aberration-corrected JEM ARM-300F operated at 300 kV. The XAFS spectra data were collected at 1W1B and 1W2B station in Beijing Synchrotron Radiation Facility.

### Electrochemical study

Electrochemical studies were performed in an electrochemical flow cell, as reported in our previous work^31^. The dimension of the gas chamber, cathodic chamber, and anodic chamber were the same, which was 1.5 × 0.7 × 1 cm^3^. The distance between the electrode and the membrane was 1 cm and the electrode area was 1.05 cm^2^. The silicone gaskets (1 mm) were placed between each component for sealing and the device was tightened using four bolts. To construct the cathode electrode, 1 mL of methanol solution containing 10 mg of obtained Cu catalysts and 30 µL of Nafion ionomer solution (5 wt% in H_2_O) was first mixed and sonicated. Next, the catalyst slurry (0.3 mL) was sprayed onto the gas diffusion layer (GDL, YLS-30T) by the airbrush. The Ni foam was used as the counter electrode. The anodic and cathodic chambers were separated by an anion exchange membrane (FumasepFAA-3-PK-130). The electrolysis was performed using a CHI 660e electrochemical workstation equipped with a high-current amplifier CHI 680c. The Ag/AgCl electrode was used as the reference, and it was calibrated with respect to RHE: E (versus RHE) = E (versus Ag/AgCl) + 0.197 V + 0.0591 V/pH×pH. Furthermore, the iR compensation was carried out automatically at the constant potential using the function “iR Comp Test Results” in the electrochemical workstation, and 85% ohmic resistance correction was applied in all the measurements. During the CO_2_RR, the electrolyte for both the cathode and the anode was 1 M KOH. The flow rate of CO_2_ gas was 20 sccm. The gaseous and liquid products of electrochemical experiments were analyzed by gas chromatography (GC, HP 4890D), ^1^H NMR (Bruker Avance III 400 HD spectrometer), respectively.

The FE of the product was computed from:

$${{{\mbox{FE}}}}=\frac{{{{\mbox{moles}}}} \,{{{\mbox{of}}}} \,{{{\mbox{product}}}}}{{{{\mbox{Q}}}}\,/ \,{{{\mbox{nF}}}}} \times 100\%$$

in which n is the number of electrons required to generate the product, F the Faraday constant and Q charge.

### ECSA measurement

ECSA was measured by Double-layer capacitance and Pb underpotential deposition methods. For the Double-layer capacitance method, the CV scans were conducted at the potential ranging from 0.2 V to 0.1 V vs. RHE. The scan rates were 20, 30, 50, 80, 100, and 120 mV s^−1^. The capacitance currents at 0.15 V vs. RHE were plotted against the scan rates. For the Pb underpotential deposition methods, an N_2_-saturated solution of HClO_4_ (100 mM) and Pb(ClO_4_)_2_ (1 mM) was used as the electrolyte. The cathode was held at −0.081 V vs RHE for 60 s and then CV was recorded between −0.5 and 0.2 V vs RHE at 5 mV s^−1^.

### Operando XAS measurements

The XAFS spectra data were collected at 1W1B and 1W2B station in Beijing Synchrotron Radiation Facility using a custom-designed flow cell. The Ag/AgCl electrode and Ni foam were used as the reference electrode and counter electrode. The electrolyte for both the cathode and the anode was 1 M KOH. The different potentials were applied during the Operando XAS measurements, and a 15-min electrolysis was performed at each potential before the signals were collected. The acquired EXAFS data were processed according to the standard procedures using the Athena and Artemis in the IFEFFIT software packages. Firstly, the post-edge background was subtracted and the spectra was normalized. Subsequently, the χ(k) data were Fourier transformed to real (R) space using a hanning windows (dk = 1.0 Å^−1^) to separate the EXAFS contributions from different coordination shells. The least-squares curve parameter was fitted by using the ARTEMIS module of the IFEFFIT software packages.

### In situ SERS measurements

The measurements were carried out using a Horiba LabRAM HR Evolution Raman microscope with a common objective (10×) in a modified flow cell, and the 785-nm laser was chosen. The Ag/AgCl electrode and Ni foam were used as the reference electrode and counter electrode. The electrolyte for both the cathode and the anode was 1 M KOH. The different potentials were applied during the In situ SERS measurements, and a 15-minute electrolysis was performed at each potential before the signals were collected.

## Supplementary information

Supplementary information

Description of Additional Supplementary Files

Supplementary Movie 1

Supplementary Movie 2

Supplementary Movie 3

Supplementary Movie 4

Supplementary Movie 5

Supplementary Movie 6

## Data Availability

The data supporting the findings of this work are available within the article and its Supplementary Information files. All the data reported in this work are available from the authors on reasonable request.
