# Supplementary material for: Oxidation of metallic Cu by supercritical CO2 and control synthesis of amorphous nano-metal catalysts for CO2 electroreduction
Source: Nat Commun. 2023 Feb 25;14:1092. doi: 10.1038/s41467-023-36721-8 (PMC9968285; doi:10.1038/s41467-023-36721-8)
Supplement: Supplementary file 1 — Supplementary information [file 41467_2023_36721_MOESM1_ESM.pdf]

Oxidation of metallic Cu by supercritical CO_2 and control synthesis of amorphous nano-metal catalysts for CO_2 electroreduction

Chunjun Chen,[1,2] Xupeng Yan,[1,2] Yahui Wu,[1,2] Xiudong Zhang,[1,2] Shoujie Liu,[3] Fanyu Zhang,[1,2] Xiaofu Sun[1,2] Qinggong Zhu[1,2] Lirong Zheng,[4] Jing Zhang,[4] Xueqing Xing,[4] Zhonghua Wu,[4] and Buxing Han[1,2,5,6]*

1. Beijing National Laboratory for Molecular Sciences, Key Laboratory of Colloid and Interface and Thermodynamics, Institute of Chemistry, Chinese Academy of Sciences, Beijing 100190, China.
2. School of Chemistry and Chemical Engineering, University of Chinese Academy of Sciences, Beijing 100049, China.
3. Chemistry and Chemical Engineering of Guangdong Laboratory, Shantou 515063, China.
4. Institute of High Energy Physics, Chinese Academy of Sciences, Beijing 100049, China.
5. Physical Science Laboratory, Huairou National Comprehensive Science Center, No. 5 Yanqi East Second Street, Beijing 101400, China
6. Shanghai Key Laboratory of Green Chemistry and Chemical Processes, School of Chemistry and Molecular Engineering, East China Normal University, Shanghai 200062, China

Computational Method

1) Machine learning details

Mechanisms for the amorphization can be studied by ab initio molecular dynamics (AIMD). However, AIMD is prohibitively expensive. A compromise is the introduction of a neural network (NN). It represents functions of many variables in a continuous way and interpolates within the training set, which allow to obtain a faithful representation of the ab initio potential energies and forces, at a much reduced cost.[S1] Practically, NNs based MD is an "equivalently accurate, but faster" AIMD. Till today, the structure of NN used to represent DFT result has gradually reached a paradigm, which is the high-dimensional neutral network potentials (HDNNPs) proposed by Behler and Parrinello.[S1, S2] It decouples the total energy of the system to a sum of atomic energies. Using the concept of "nearsightedness" to regard the atomic potential as the functionals of the local chemical environment up to a cutoff radius and are computed by individual atomic neural networks.

In this paper, to simulate the amorphization mechanism, we also introduced such HDNNPs. To compose appropriate studying set for machine learning, we built both surface and bulk structures of Cu. The surface structures contained Cu (111) surface and (111x111) step surface. For both of them, models with and without CO_2 were built, and CO_2 with a density of 0.316g/cm^3 was chosen as the data set, which is similar to that of the SC CO_2. So we have bulk Cu, Cu empty surface x2 (111&111x111), Cu-CO_2 interface x2 (111&111x111) and dense CO_2, having 6 structures in total. For each structure, we used DFTMD to generate DFT data set (details for the parameters chosen are given in the section of DFT and DFTMD details). Three temperatures, namely, 300, 900 and 3000 K were chosen, with 500 data generated for each temperature. Therefore, we obtained 9000 DFT data in total to train the NN.

Among the many NN-based methods for constructing accurate force fields, we choose here the DeePMD-kit package.[S3] The codes achieve a high-dimensional neutral network that preserves all the natural symmetries during structure analysis. In a meanwhile of efficiently repeating indistinguishable with the DFT based data, it will generate

a small volume of the force field. During the learning process, the cut-off radius is set to be 5 Å. The maximum number of neighbor atoms that are treated by full relative coordinates are set as 50, 32 and 32 for Cu, C and O. The smoothing is set to begin from 1/5 to 2. The number of neurons in each hidden layer numbers of the embedding nets are 10, 20 and 40. And that of hidden layer numbers of the fitting net are 240, 240 and 240. The perfectors of energy and force loss at the start of the training are set to be 0.02 and 1000, while that at the limit of the training are set to be 2 and 1. The starting learning rate, the decay step and the decay rate are set as 0.001, 1000 and 0.95.

2). DFT and DFTMD details

All the DFT calculations are implemented via the Quantum Espresso.[S4] Spin-polarized DFT calculations were performed with periodic super-cells under the generalized gradient approximation (GGA) using the Perdew-Burke-Ernzerhof (PBE) functional for exchange-correlation and the ultrasoft pseudopotentials for nuclei and core electrons. The Kohn-Sham orbitals were expanded in a plane-wave basis set with a kinetic energy cutoff of 30 Ry and the charge-density cutoff of 300 Ry. The Fermi-surface effects were treated by the smearing technique of Methfessel and Paxton, using a smearing parameter of 0.02 Ry. For all of the structures, the convergence criteria are set as 10^{-4} Ry/Bohr of Cartesian force components acting on each atom and 10^{-4} Ry of total energy. For surface structures, the k-point are set to be 3x3x1, while that of bulk structure is 3x3x3.

For DFTMD calculation, the time step is set to be 1 fs. The canonical ensemble condition was imposed by a Nose thermostat with target temperatures (300, 900 and 3000 K). All of the structures are performed by 100 fs. All the snapshots are recorded for NN training.

3). Amorphization simulation details.

i Simulation parameters

The synthesis simulation is done by large-scale atomic/molecular massively parallel simulator (LAMMPS),[S5] with the force field generated by the DeePMD-kit. During the simulation, we put six hard plates with the expressions

of z=0&100, x=0&100 and y=0&100 to set the boundary of the atoms; and we use a Morse potential to describe the interaction between these plates and the atoms near them, so that every atom approaches such plate will rebound by the Morse potential. Since the Morse potential is large and has a truncation distance of 6 Å, the actual activity space for atoms is x ∈ [6, 94], y ∈ [6, 94] and z ∈ [6, 94] (unit: A). This gives the cubic of 8.8 x 8.8 x 8.8 nm. Additionally, we have included a gravity for all the atoms, with the direction of [-1, -1, -1]. This means that if the temperature is low enough, all the atoms will gradually gather at one corner of the box. We do this to increase as large as possible the contact between Cu and CO_2, otherwise the drop of CO_2 will suspend in the box.

ii Temperature chosen

An important obstacle for the amorphization simulation is the paradox between the long experiments time (forming 2 nm of amorphous Cu in 4 h) and the short simulation time (0.1 ns /per day for 16 core's CPU). To overcome this, we have to use high temperature. However, it is important to ensure this high temperature will still retain the property at low temperature. Therefore, we use Arrhenius as the criteria: if such applied temperature can still follow Arrhenius equation fitted at low temperature, we would say that such temperature is accessible.

Specifically, since amorphization is about diffusion of O atoms inside the lattice of Cu, we use diffusion coefficient (D, calculated by mean squatted displacement, MSD) as the property to test its relation with temperature T. If D can scale with exp(-1/T) up to low temperature (e.g., 600 K), the associated temperature will be regarded as accessible.

For this, we created a small cubic bulk of Cu (3x3x3 nm^3) and replace the 30 inner Cu into O atoms. During the simulation, the pressure is set as 8 MPa, which is the same as that in experiments. Based on such model, we tested the temperature from 600 to 1700 K, with the results shown in Supplementary Fig. S13. We found a good linear relation from 600 K to 1300 K. This indicates that in these temperature scale, the diffusion fits well with the Arrhenius equation. And this means that there is no phase transition for such temperature. Consistently, for

temperature of 1700 K, we see the melting of Cu, where the lattice of Cu is mixed up. This is the reason why D at this temperature is much larger than the line fitted with Arrhenius equation. Besides, at temperature lower than 600 K, e.g., 400 K, the diffusion frequency is so low that we cannot sample enough diffusion process (only one time in 0.4 ns), and its value is not accurate. This again confirms the necessity to increase the temperature. Among these temperatures, we found that 1300 K is a good candidate, because in such temperature, Cu lattice can be kept well during the simulation time (notice despite it exceeds the melting point of Cu, the high pressure keeps it from melting). So we fix Cu temperature at 1300 K.

Furthermore, at such temperature, we consider that we can further increase the temperature of O. We found that from 400 K to 13000 K, the diffusion coefficient and the temperature also follows the Arrhenius equation. So we choose temperature of 9000 K for C and O during the Amorphization simulation.

iii Amorphization simulation

The synthesis simulation is done by large-scale atomic/molecular massively parallel simulator (LAMMPS),[S6] with the force field generated above. The simulation was carried out inside a rigid cubic box (that will not expand or shrink during the simulation, and meanwhile bouncing off the atoms that hit it) that has the edge length of 8.8 nm. Inside this box we put a Cu with a regular octahedron that possessed a length of 4.2 nm. Besides Cu, we put inside 3000 CO_2 molecules, which would result in a CO_2 density of 0.316 g/cm^3. This density matched well with the density of a SC CO_2. During the simulation, the system was first equilibrated under canonical ensemble condition (NVT) imposed by a Nosé thermostat with a target temperature of 300 K for 20 ps. Then we use a high temperature to overcome the too slow amorphization process in experiments (forming 2 nm of amorphous Cu in 4 h). Specifically, based on the test above, we chose 9000 and 1300 K for CO_2 molecules and Cu, respectively. We kept such temperature for 120 ps. Then, to simulate the C and O departure during the decompress and electrochemical reduction, we then execute the simulation that will delete the C and O atoms once they run outside

a circle. Such circle is set to be centered at the barycenter of all the Cu atoms and has a radius of 3.0-1.7 nm (the radius decrease uniformly with time). We check whether to delete C and O atoms every 50 fs. After 50 ps, we got the rudiment of the amorphous Cu. Finally, such achieved rudiment was put in thermostat of 300 K for another 50 ps to get the final amorphous Cu (attached file of *movie 1).

4). Activity study by the cut model

After obtaining the above-mentioned model, it is necessary to discuss the activities on the surface sites. To do this, we first randomly pick 40 surface sites, then cut the sites along with their neighbor atoms with a cutoff radius of 4 Å (file *movie 6 for the building of cut model). Such cluster is put in the center of a periodic box with 10x10x10 Å^3. We make these cut-off models to simulate the impact from the selected site, as well as its surroundings. The associated structure is given in attached file named *movie 6. When implementing DFT calculations on these clusters, half of the atoms on the bottom are fixed in order to keep the structures in the amorphous Cu.

5). Details for calculating CO adsorption energies and the associated CO coverage.

Adsorption energy, ΔE_{*A}, is defined as $\Delta E_A = E_{*A} - E_{*} - E_A$, where E_{*A}, E_{*} and E_A are defined as the total energy of adsorption model (base+ adsorbate), base and gas-phased adsorbate. In particular, * stands for the catalytic sites. E_{*A}, E_{*} and E_A can all be calculated by DFT. In this paper, our goal was to calculate the difference of ΔE_{CO} between Cu111 and amorphous Cu. So we chose to use the relative value of ΔE_{*CO} vs Cu111. That is:

$$\Delta E_{*A}^{M_{ref}} = \Delta E_{*A}^{M} - \Delta E_{*A}^{Cu111} \quad \text{(S1)}$$

M stands for the studied site.

To calculate the potential dependent coverage, the computational hydrogen electrode (CHE) method proposed by Norskov et al was introduced.[S9] At the potential that we are interested (-0.25 to -0.35 V), there are two reaction that are important. Namely:

$H^{+}+e+* \rightarrow *H$ (RS1)

$CO_2+2H^++2e+^*\rightarrow{}^*CO+H_2O$ (RS2)

where * stands for the reaction site. This means that there are two kinds of key adsorbates, *H and *CO on the surface. To calculate the CO coverage, we assume that these two reactions are all under equilibrium at the associated potential. Thus we have

$$u_{*H}-u_{*}-u_{H+}-u_{e}+RTln\frac{\theta_{*H}}{1-\theta_{*H}-\theta_{*CO}}=0 \quad \text{(S1a)}$$

$$u_{*CO}-u_{*}-2u_{H+}-u_{CO_2}-2u_{e}+RTln\frac{\theta_{*CO}}{1-\theta_{*H}-\theta_{*CO}}=0 \quad \text{(S1b)}$$

Where u_{*A} represents the chemical potentials for specie A. Its value equates the DFT based energy pluses the contribution from zero point energy and entropy. Eq.S1a subtracts Eq.S1b, we will get

$$u_{*\mathrm{H}}-u_{*\mathrm{CO}}+u_{e}+u_{\mathrm{CO_2}}+u_{\mathrm{H}+}+RTln\frac{\theta_{*\mathrm{H}}}{\theta_{*\mathrm{CO}}}=0 \quad \text{(S2)}$$

With the help of the CHE method, Eq.S2 can be turned into

$$u_{*\mathrm{H}}-u_{*\mathrm{CO}}-Ue+u_{\mathrm{CO_2}}+\frac{1}{2}u_{\mathrm{H2}}+RTln\frac{\theta_{*\mathrm{H}}}{\theta_{*\mathrm{CO}}}=0 \quad \text{(S3)}$$

With U the potential vs RHE. For $u_{*\mathrm{H}}$, we use a constant value of 0.04 eV that is calculated formerly on Cu211.[S7] While the value of $-u_{*\mathrm{CO}}-Ue+u_{\mathrm{CO_2}}+\frac{1}{2}u_{\mathrm{H2}}$ is calculated to be 0.20 eV on Cu111, at U=0 V vs RHE. For amorphous Cu, the values of $-u_{*\mathrm{CO}}-Ue+u_{\mathrm{CO_2}}+\frac{1}{2}u_{\mathrm{H2}}$ can then be calculated by the data in Figure 2f (to do this, we assume $\Delta E_{*CO}^{M}-\Delta E_{*CO}^{Cu111}=u_{*CO}^{M}-u_{*CO}^{Cu111}$, u_{*CO}^{M} means $u_{*\mathrm{CO}}$ for M), which is also around 0.2 eV. This means that, at the potential we are interested (-0.25 to -0.35 V), *H and *CO have occupied all the surface sites, i.e., $\theta_{*\mathrm{CO}}+\theta_{*\mathrm{H}}\approx 1$. Thus, we can combine this with Eq.S3 to calculate the relation between $\theta_{*\mathrm{CO}}$ and $\theta_{*\mathrm{H}}$. From this we obtained Figure S35.

6). The effect of pH on the calculation of CO adsorption energy.

In the framework of CHE method, the adsorption energy is independent with the pH. To demonstrate this, we can assume an electrocatalytic reaction:

$A^*+H^++e \rightarrow AH^*$ (1)

For (1), we set pH=0. And in alkaline, we set pH=14, its reaction formula becomes:

$A^{*}+H_2O+e \rightarrow AH^{*}+OH^-$ (2)

For (1), the reaction energy is written as

$\Delta E_a=\mu[AH^*]-\mu[A^*]-\mu[H^+]+\varphi_a e$ Eq1

For (2), the reaction energy is written as

$\Delta E_b=\mu[AH^*]-\mu[A^*]-\mu[H_2O]+\mu[OH^-]+\varphi_b e$ Eq2

Where φ_a and φ_b are the absolute electrode potential for pH=0 and pH=14. Since to compare acid and basic case, we should use the same overpotential, we have:

$\varphi_b=\varphi_a-0.059*14$ Eq3

Then, $\mu[H^+]$ is the chemical potential of H^+ at concentration of H^+ is 1 mol/L, and $\mu[OH^-]$ is the chemical potential of OH^- at concentration of OH^- is 1 mol/L. We then have:

$\mu[H_2O]=\mu[H^+]+\mu[OH^-]+RT\ln 10^{-14}=\mu[H^+]+\mu[OH^-]-0.059*14$ Eq4

Adding Eq.3 and 4 into Eq.1 and 2, we have:

$\Delta E_a=\Delta E_b$

Which proves pH will not impact our DFT results.

Supplementary Figures

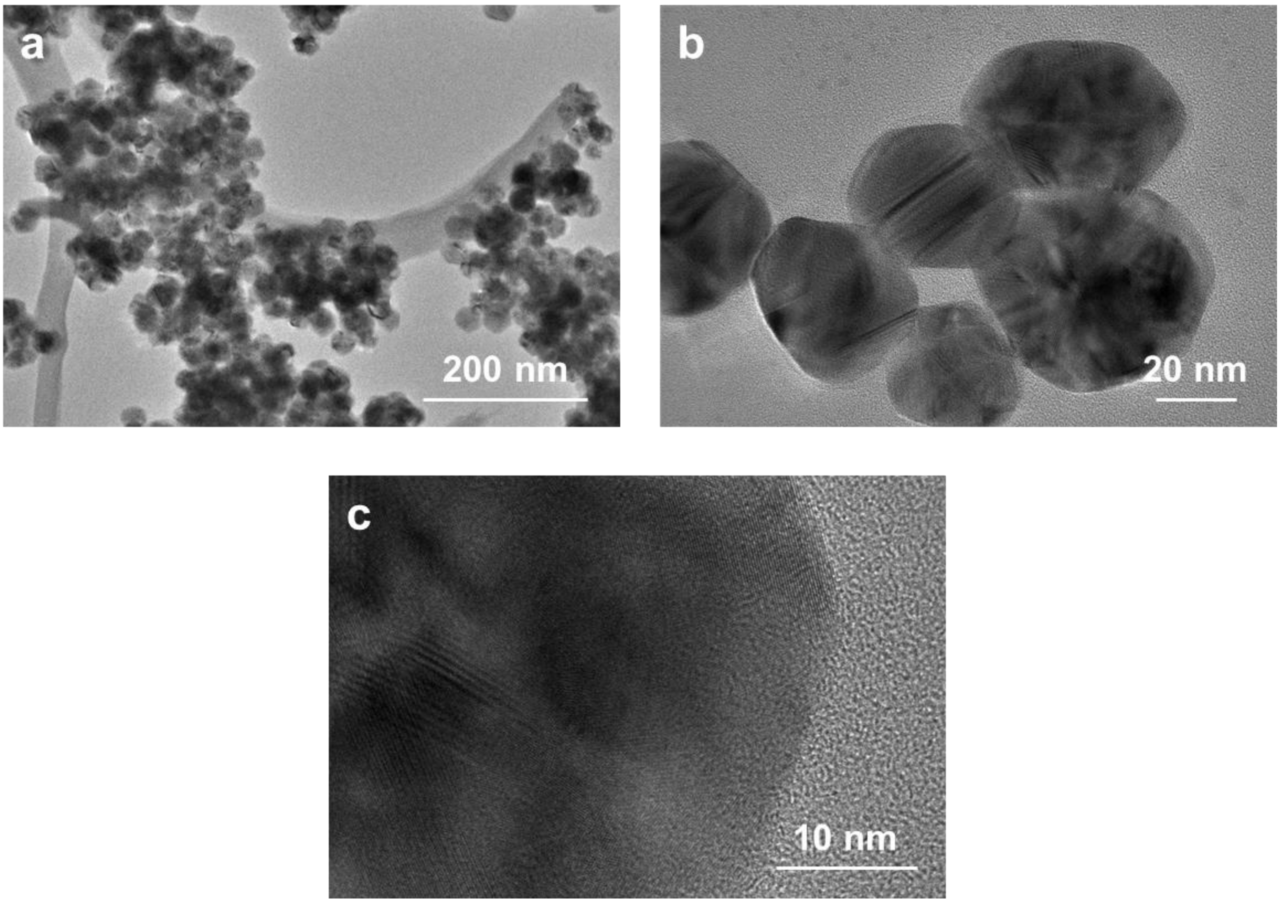

Figure S1. (a, b) The TEM images of Cu-np. (c) The HR-TEM images of Cu-np.

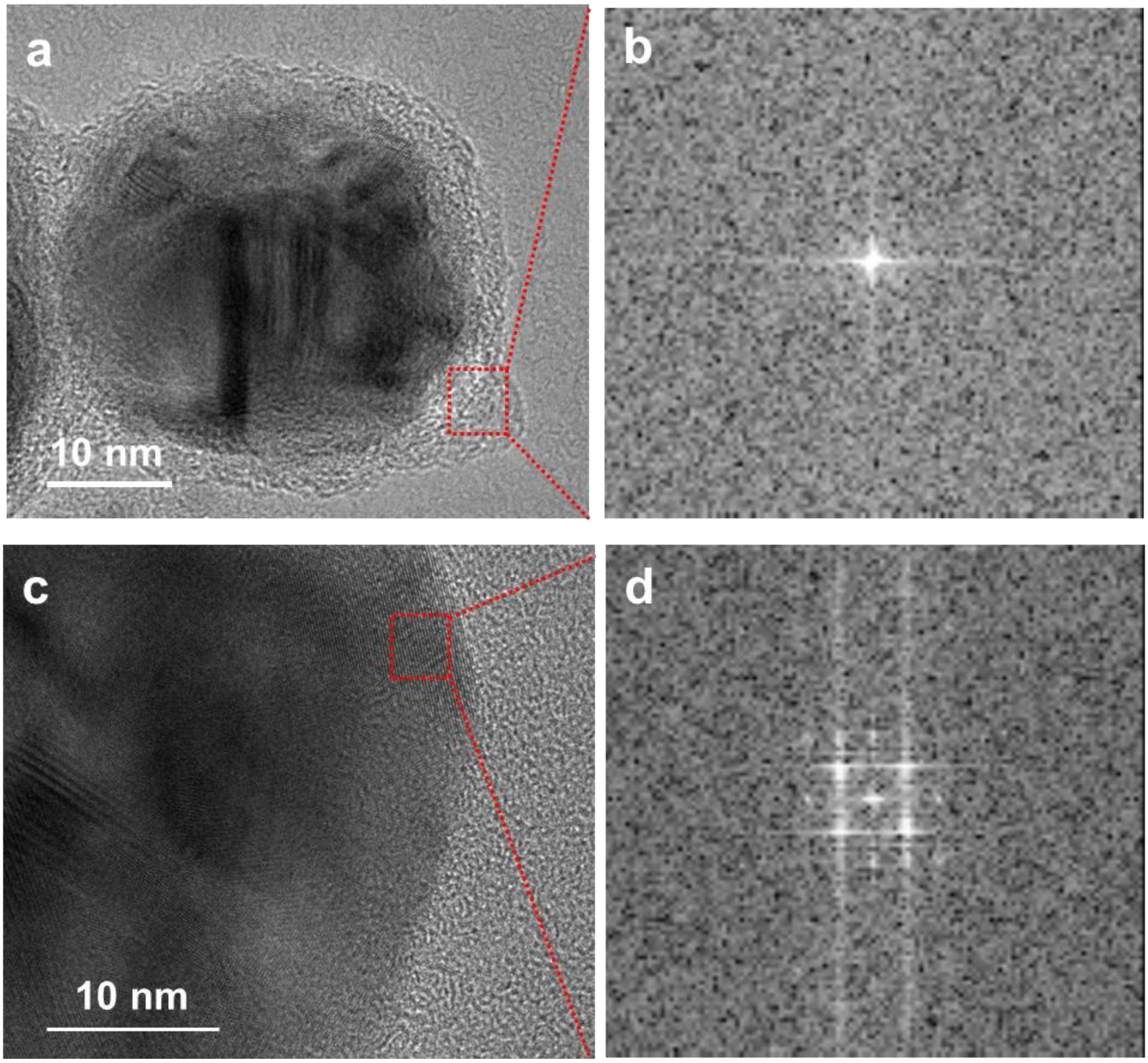

Figure S2. (a, b) The HR- TEM images of 8-Cu-12 and FFT patterns of the corresponding area in the a image. (c, d) The HR- TEM images of Cu-np and FFT patterns of the corresponding area in the c image.

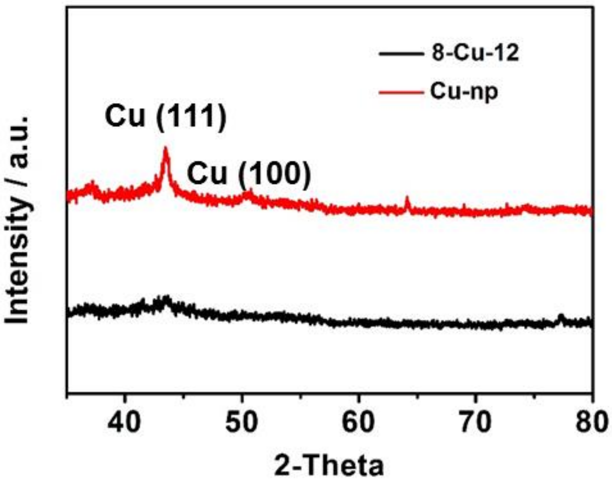

Figure S3. The XRD paterns of Cu-np and 8-Cu-12.

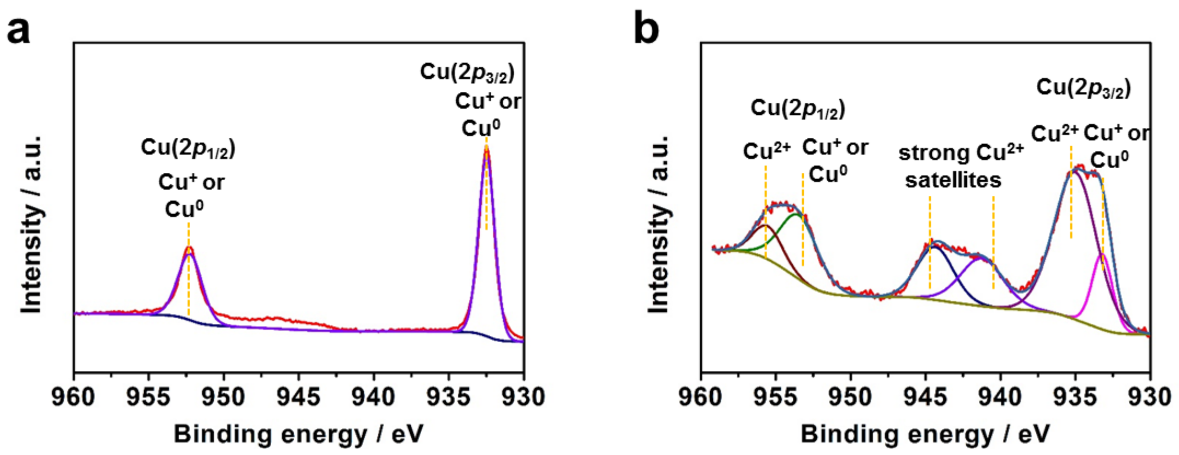

Figure S4. (a) The Cu 2*p* XPS spectra of Cu-np. (b) The Cu 2*p* XPS spectra of 8-Cu-12.

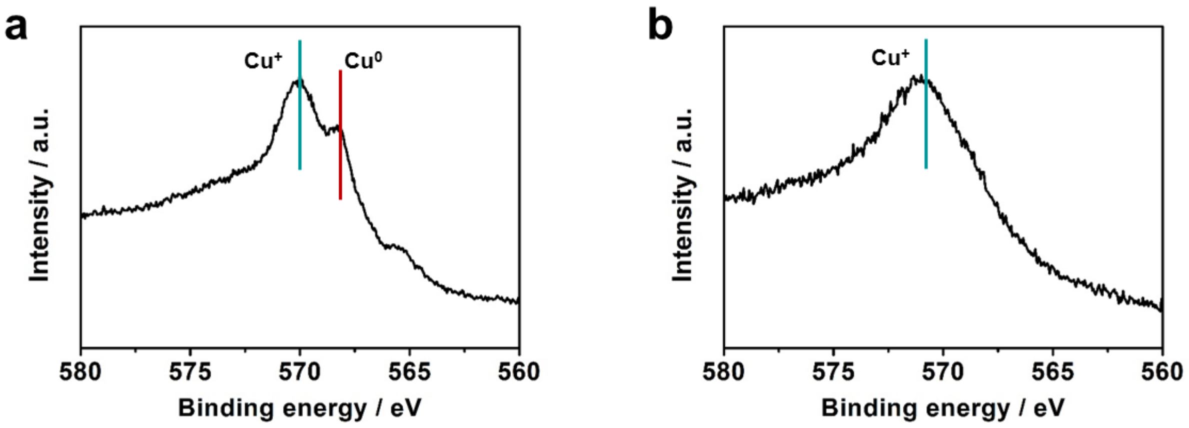

Figure S5. (a) The Cu LMN spectra of Cu-np. (b) The Cu LMN spectra of 8-Cu-12.

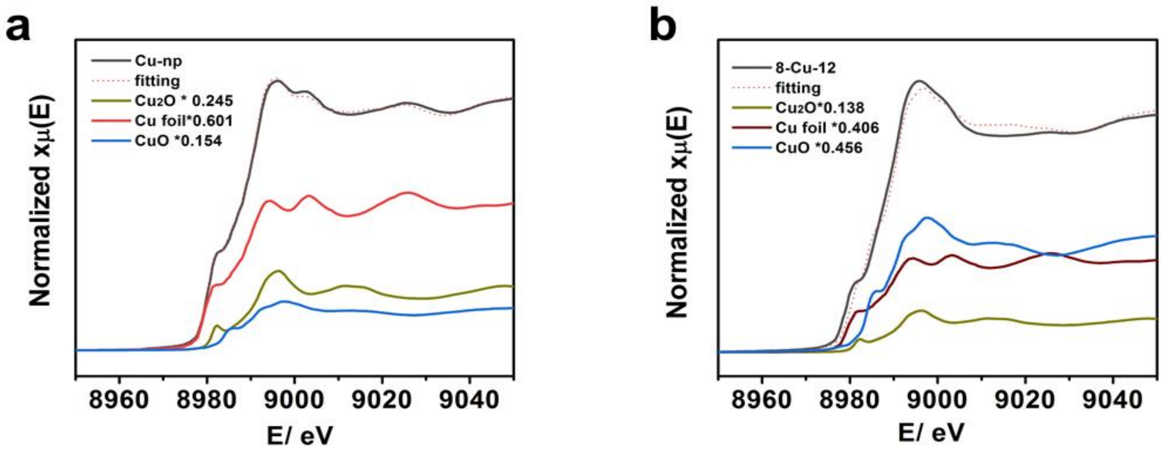

Figure S6. (a) The linear fitting of the XANES spectra of Cu-np. (b) The linear fitting of the XANES spectra of 8-Cu-12.

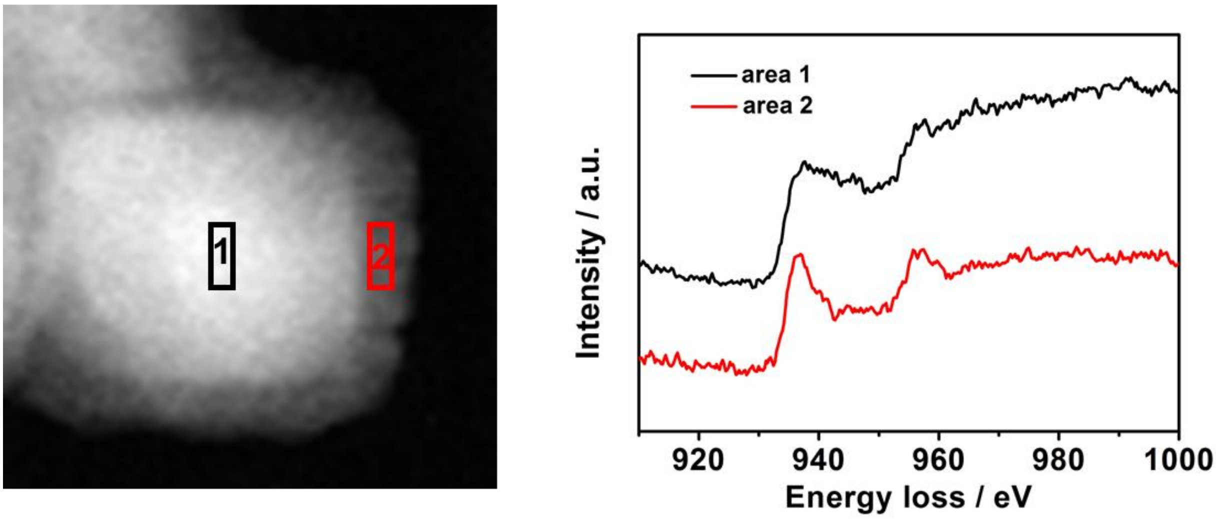

Figure S7. EELS spectra acquired from areas 1 and 2 in 8-Cu-12.

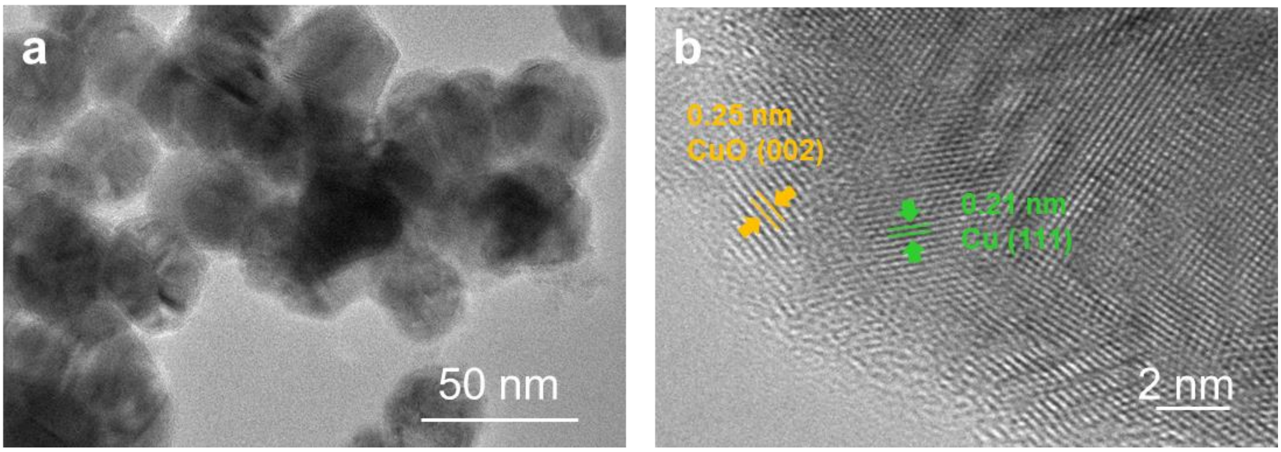

Figure S8. (a, b) The TEM and HR-TEM images of Cu-np after being oxidized by O_2 at 8Mpa.

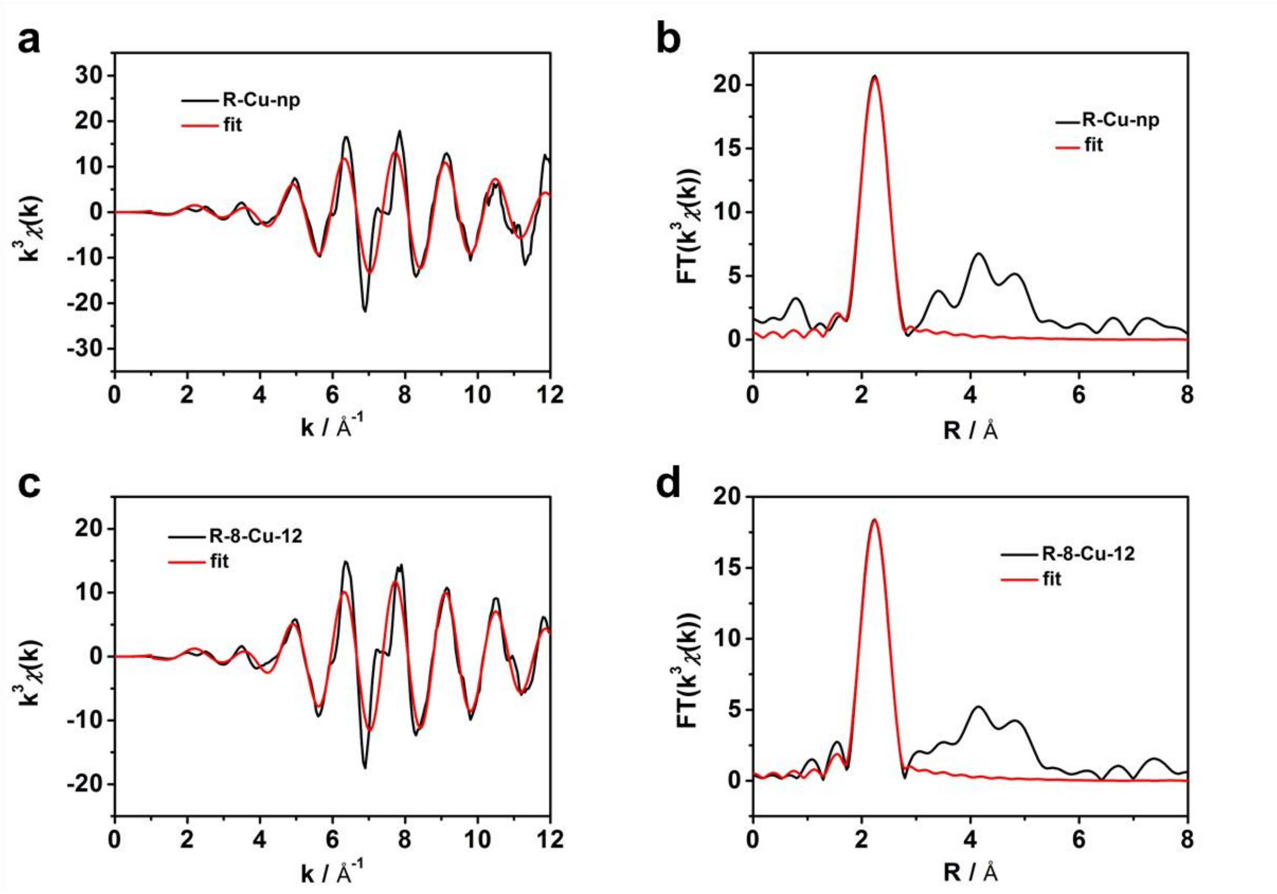

Figure S9. The EXAFS data fitting results of R-Cu-np (a, b) and R-8-Cu-12 (c, d).

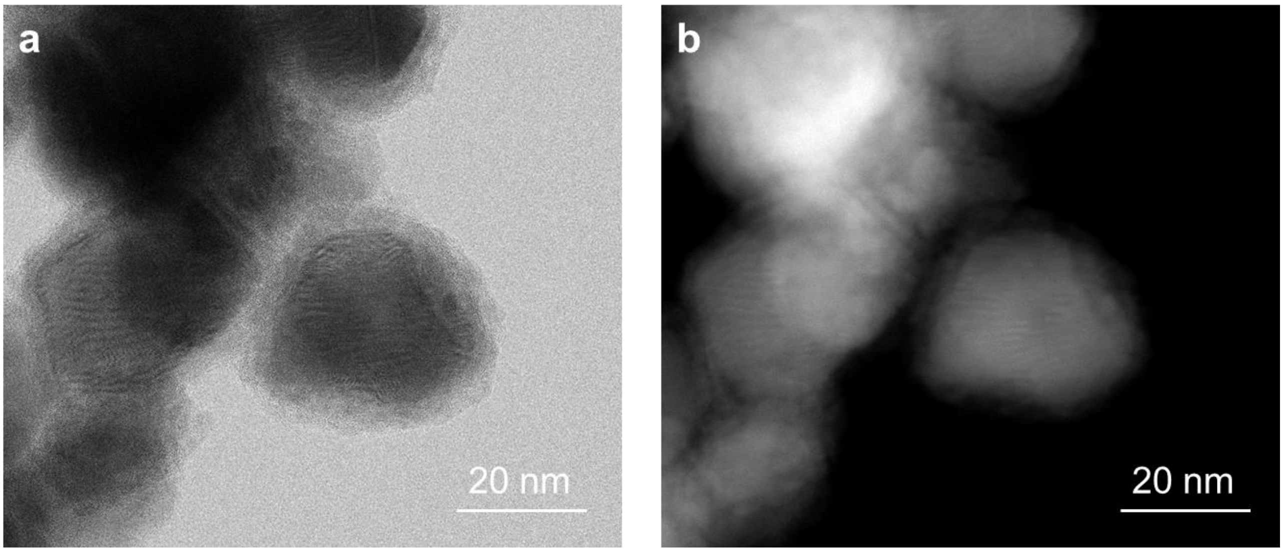

Figure S10. (a) The HR-TEM image of R-8-Cu-12. (b) The HAADF-STEM image of R-8-Cu-12.

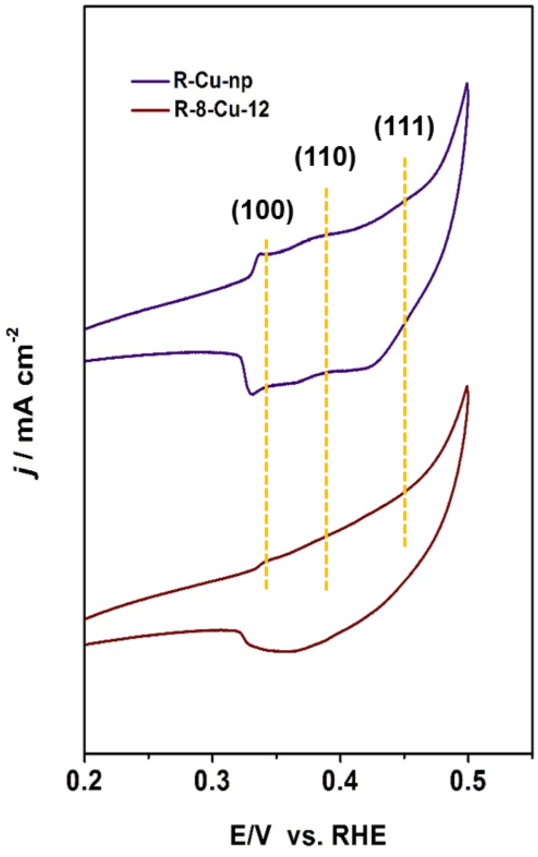

Figure S11. The Voltammograms of OH_{ads} peaks for R-Cu-np and R-8-Cu-12.

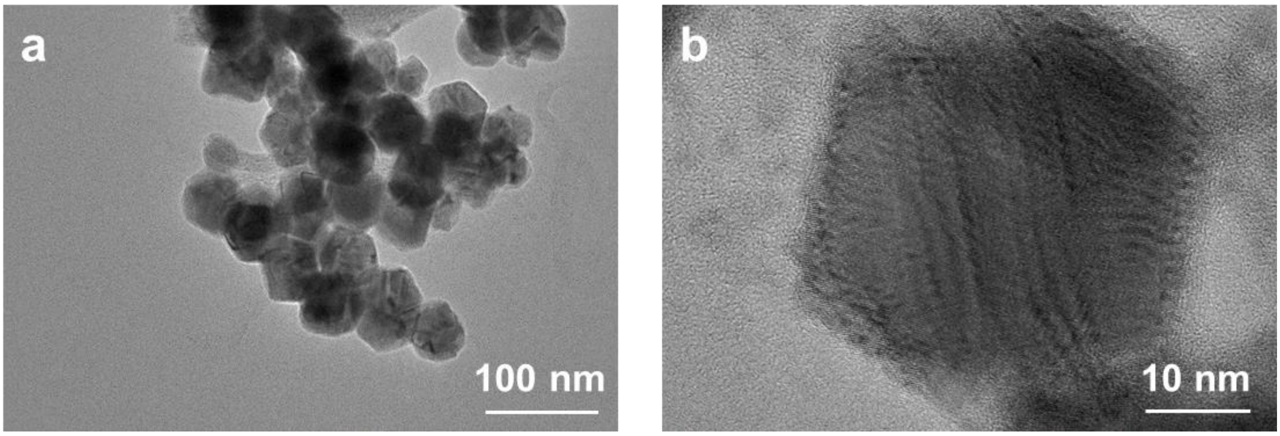

Figure S12. (a, b) The TEM and HR-TEM images of 4-Cu-12.

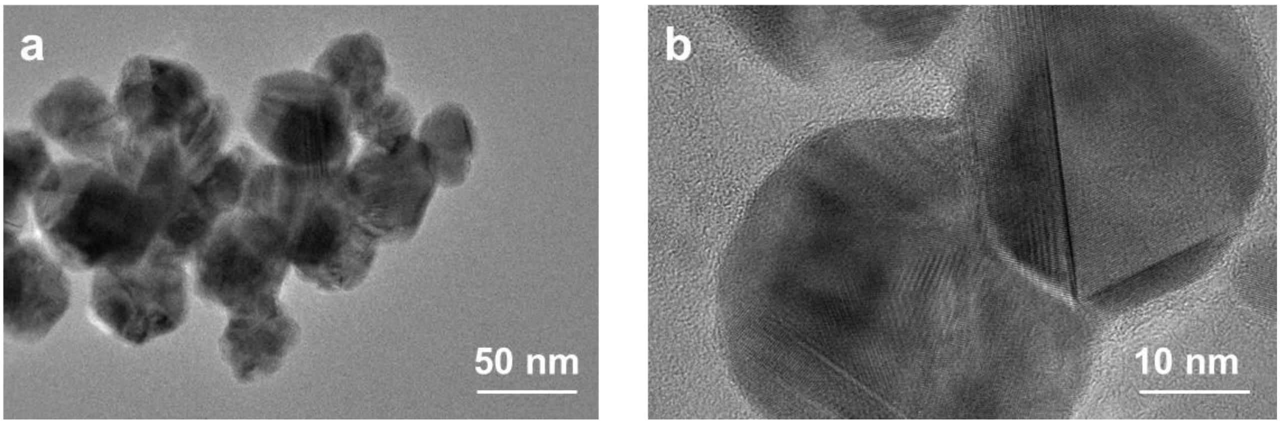

Figure S13. (a, b) The TEM and HR-TEM images of 6-Cu-12.

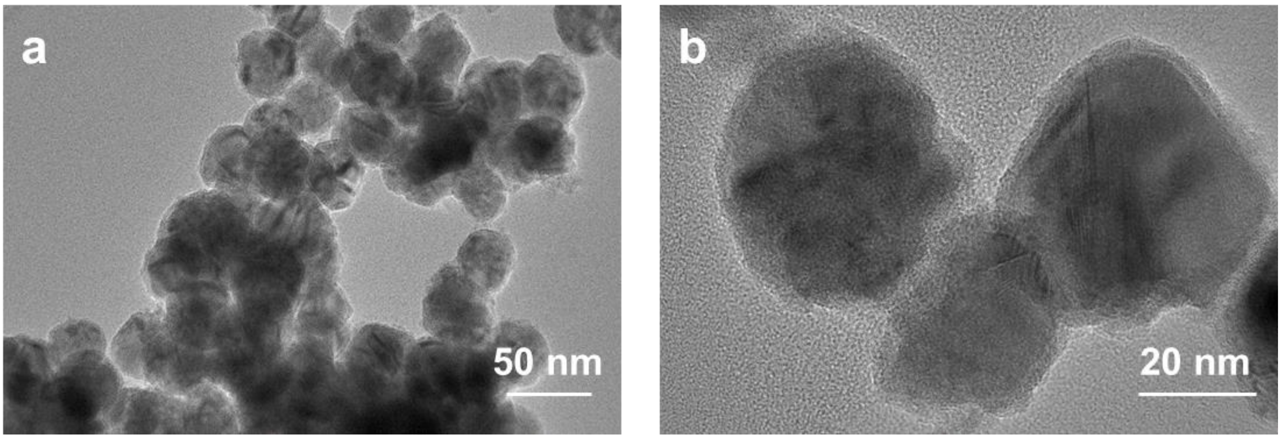

Figure S14. (a, b) The TEM and HR-TEM images of 8-Cu-4.

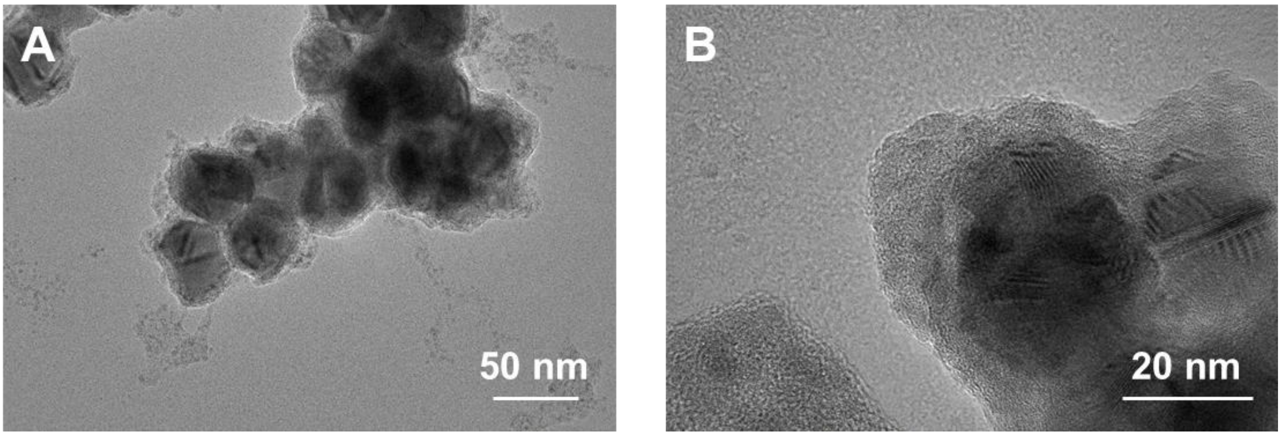

Figure S15. (A, B) The TEM and HR-TEM images of 8-Cu-16.

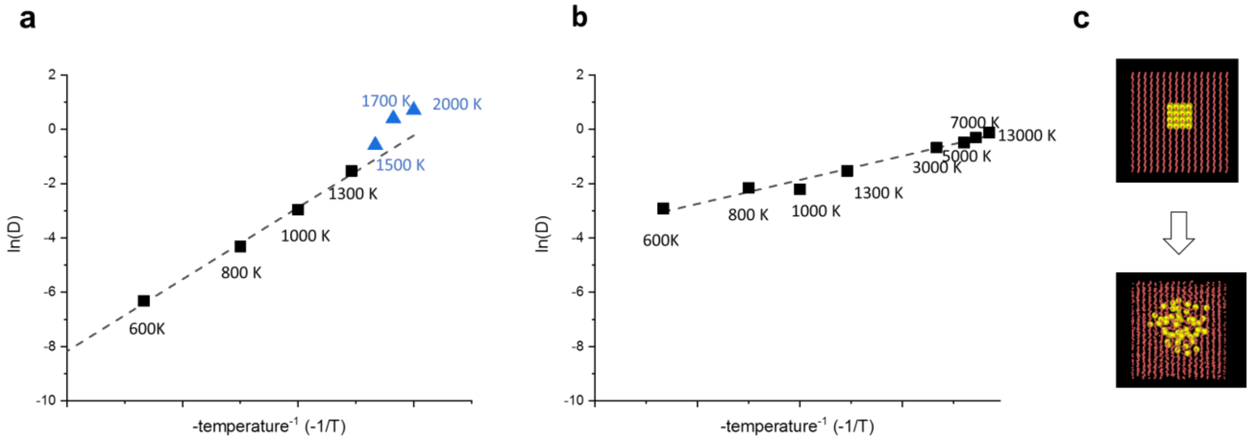

Figure S16 (a-b) The scaling relation between ln(D) and -1/T. (a) All the atoms possessed the same temperature. That is, it is the system temperature. (b) The Cu temperature is fixed at 1300 K. The temperatures are possessed by O atoms. (c) The structure used to simulate the O diffusion inside Cu lattice.

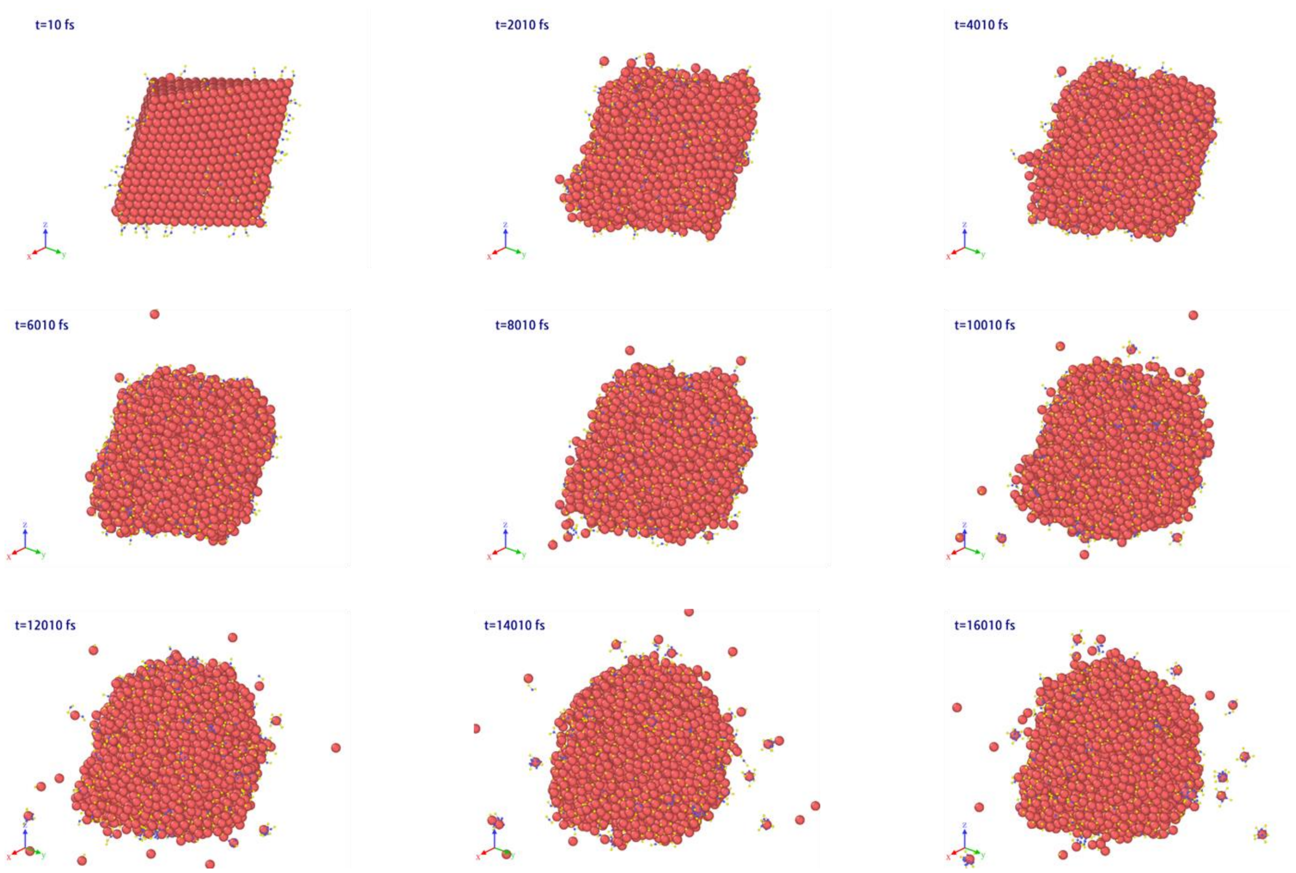

Figure S17. The amorphorization process of Cu.

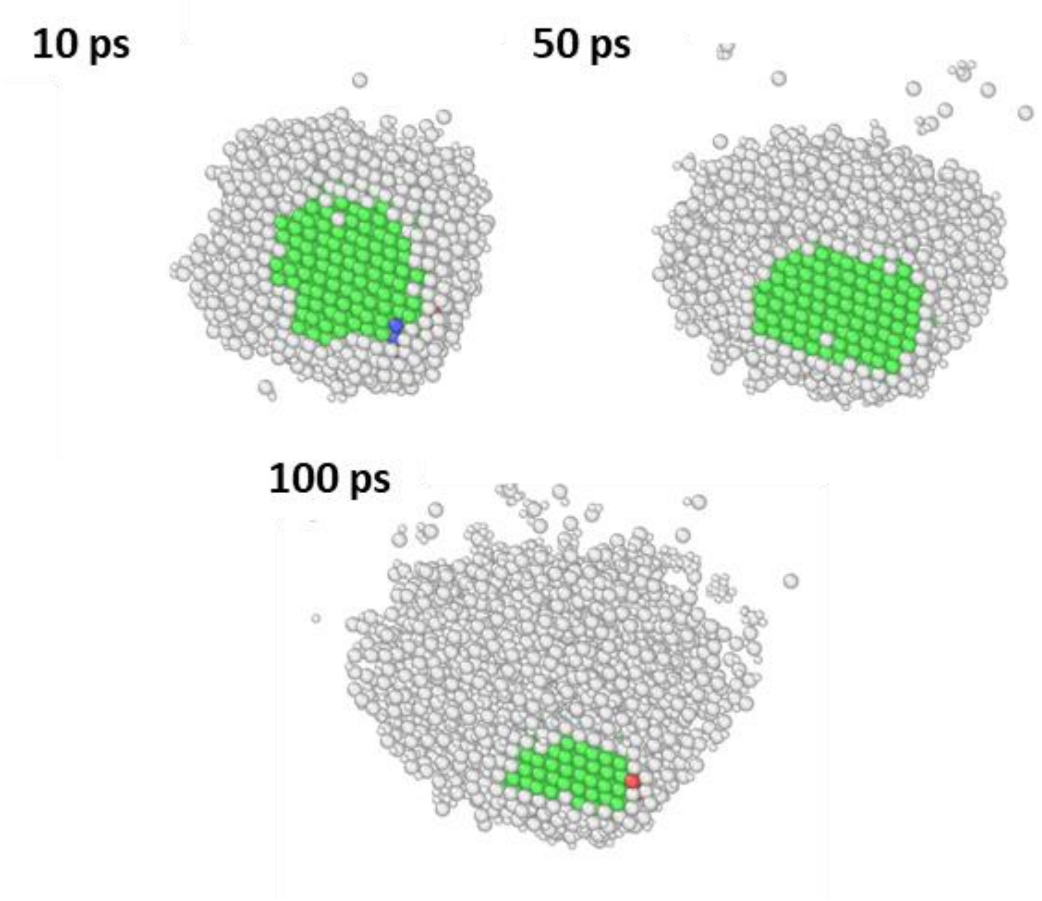

Figure S18. The decreasing fcc structures during O penetration. The green and grey portion shows the fcc and the amorphous structures, respectively. Green and red colors indicate the hcp and bcc sites. The structure identification is done based on the nearest coordination.

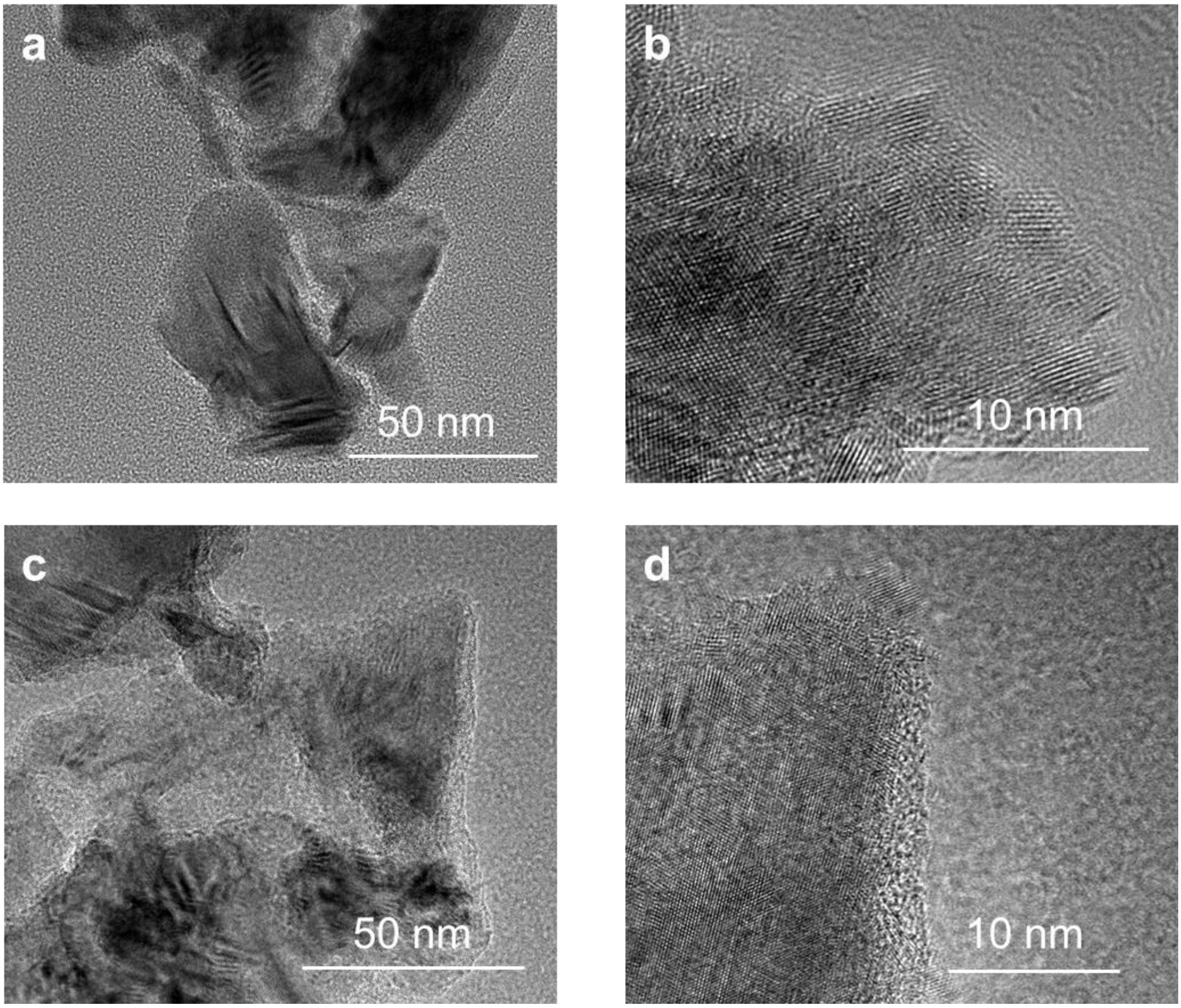

Figure S19. (a, b) The TEM and HR-TEM images of Co nanosheet. (c, d) The TEM and HR-TEM images of Co nanosheet after the SC CO_2 treatment.

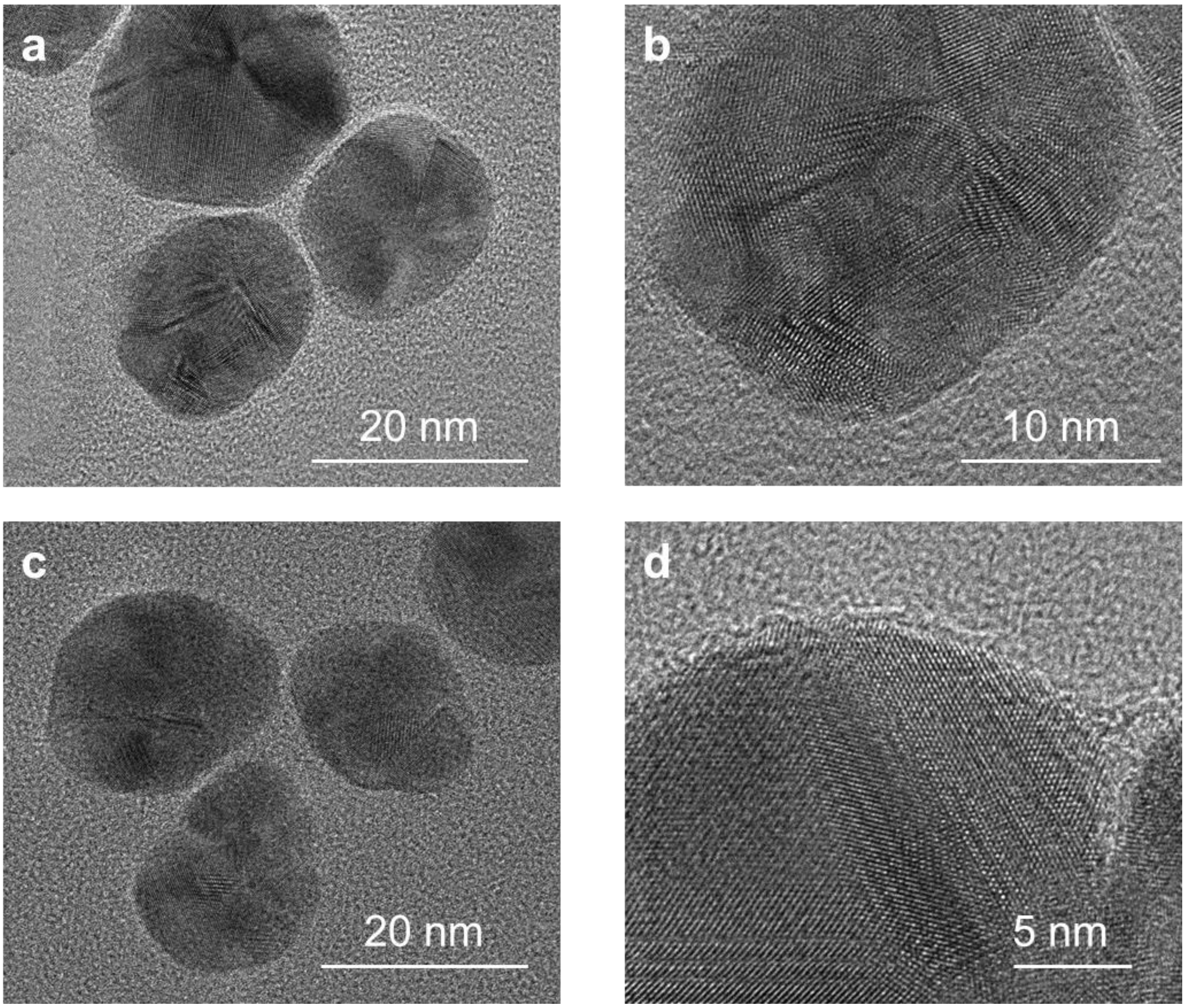

Figure S20. (a, b) The TEM and HR-TEM images of Ag nanoparticles. (c, d) The TEM and HR-TEM images of Ag nanoparticles after the SC CO_2 treatment.

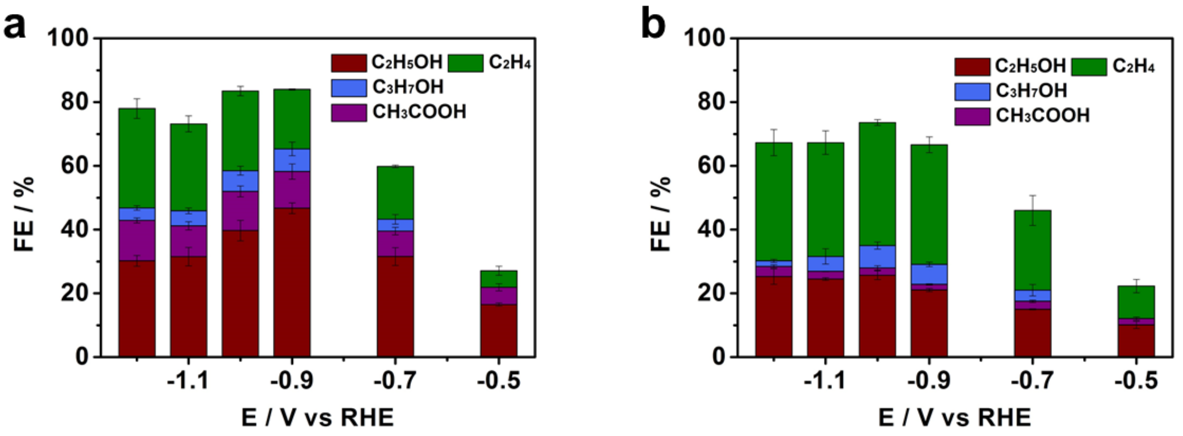

Figure S21. (a) The distribution of C2+ products over R-8-Cu-12. (b) The distribution of C2+ products over R-Cu-np. Error bars correspond to the standard deviation of three independent measurements.

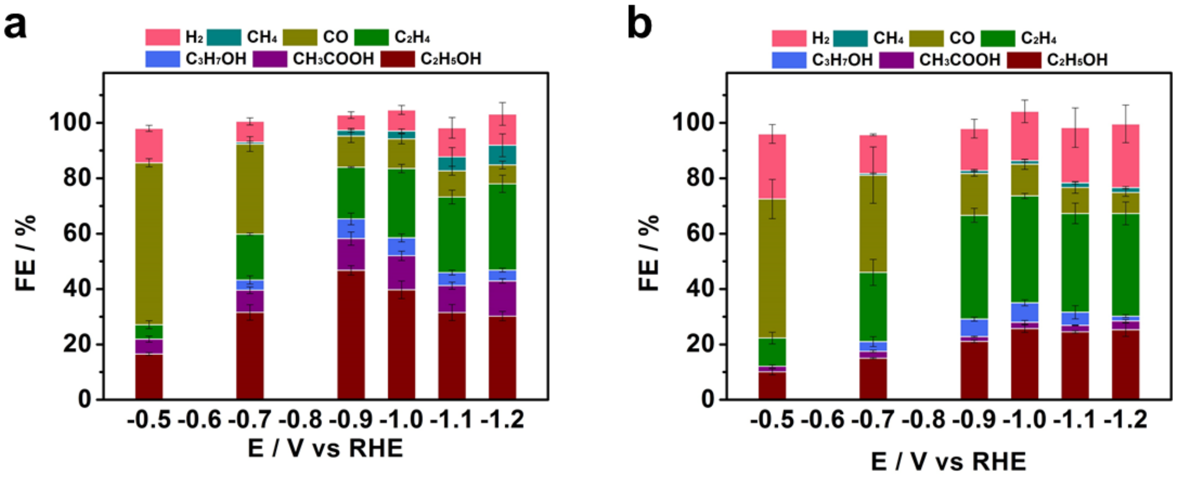

Figure S22. The distribution of H_2, C1 and C2+ products at different potentials over: R-8-Cu-12 (a) and R-Cu-np (b). Error bars correspond to the standard deviation of three independent measurements.

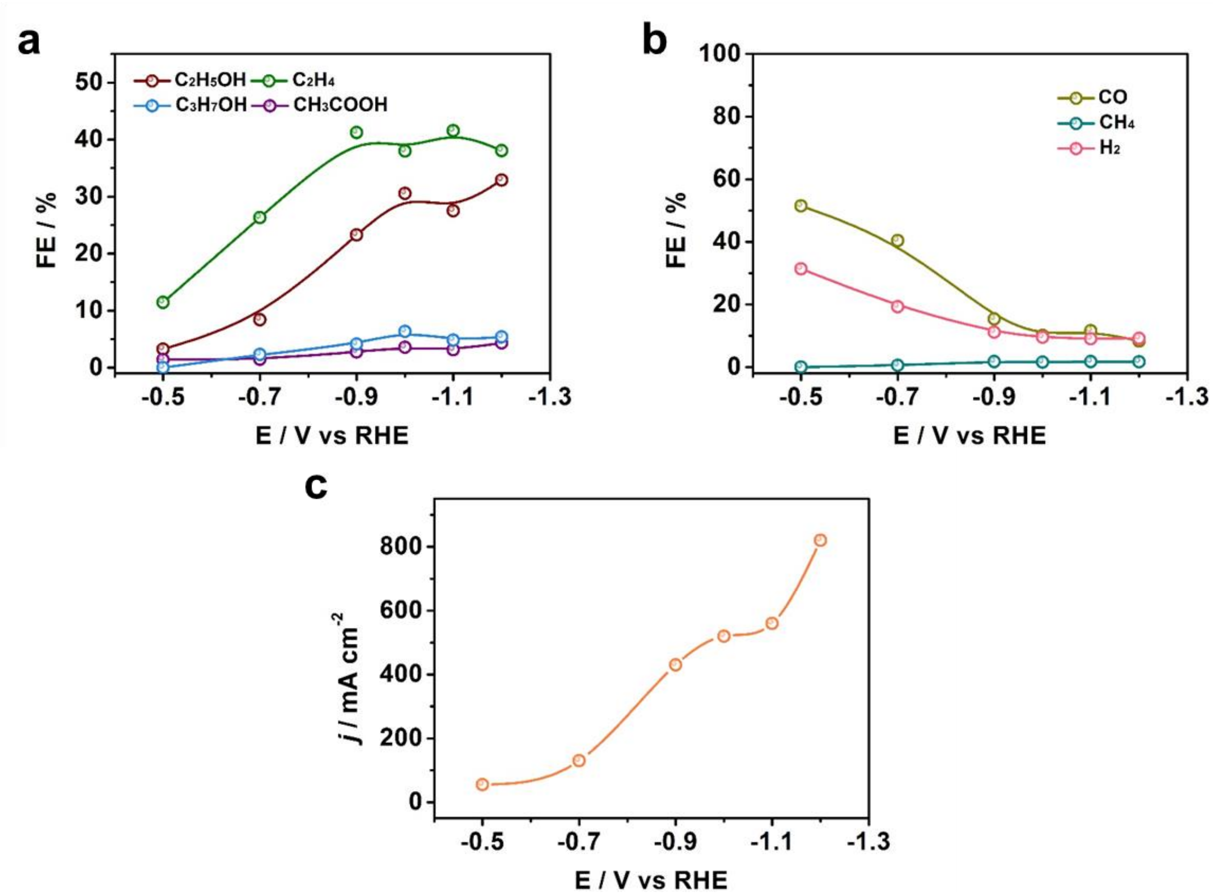

Figure S23. (a) The FE of C2+ products at different potentials over R-4-Cu-12. (b) The FE of H_2 and C1 products at different potentials over R-4-Cu-12. (c) The current density over R-4-Cu-12 at different potentials.

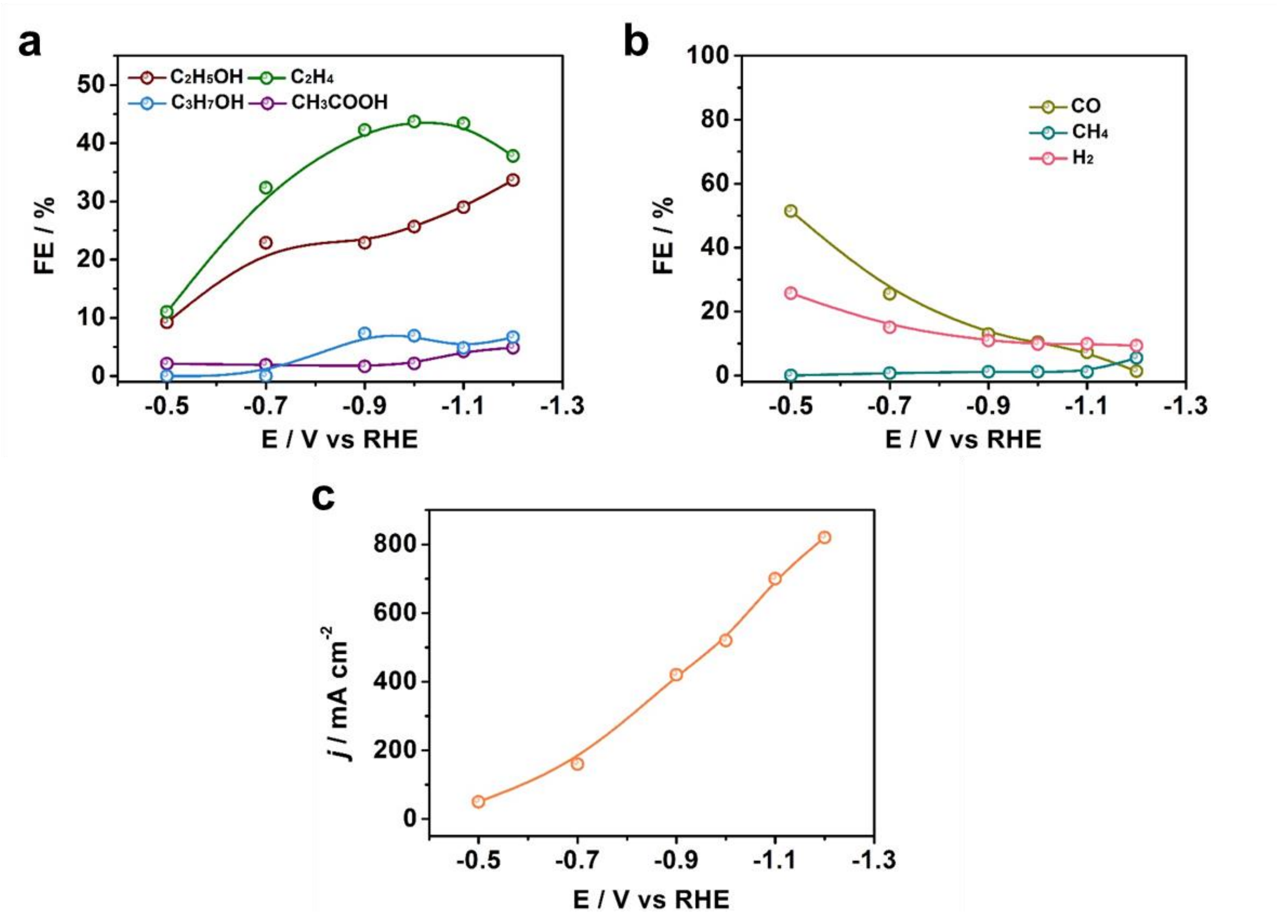

Figure S24. (a) The FE of C2+ products at different potentials over R-6-Cu-12. (b) The FE of H_2 and C1 products at different potentials over R-6-Cu-12. (c) The current density over R-6-Cu-12 at different potentials.

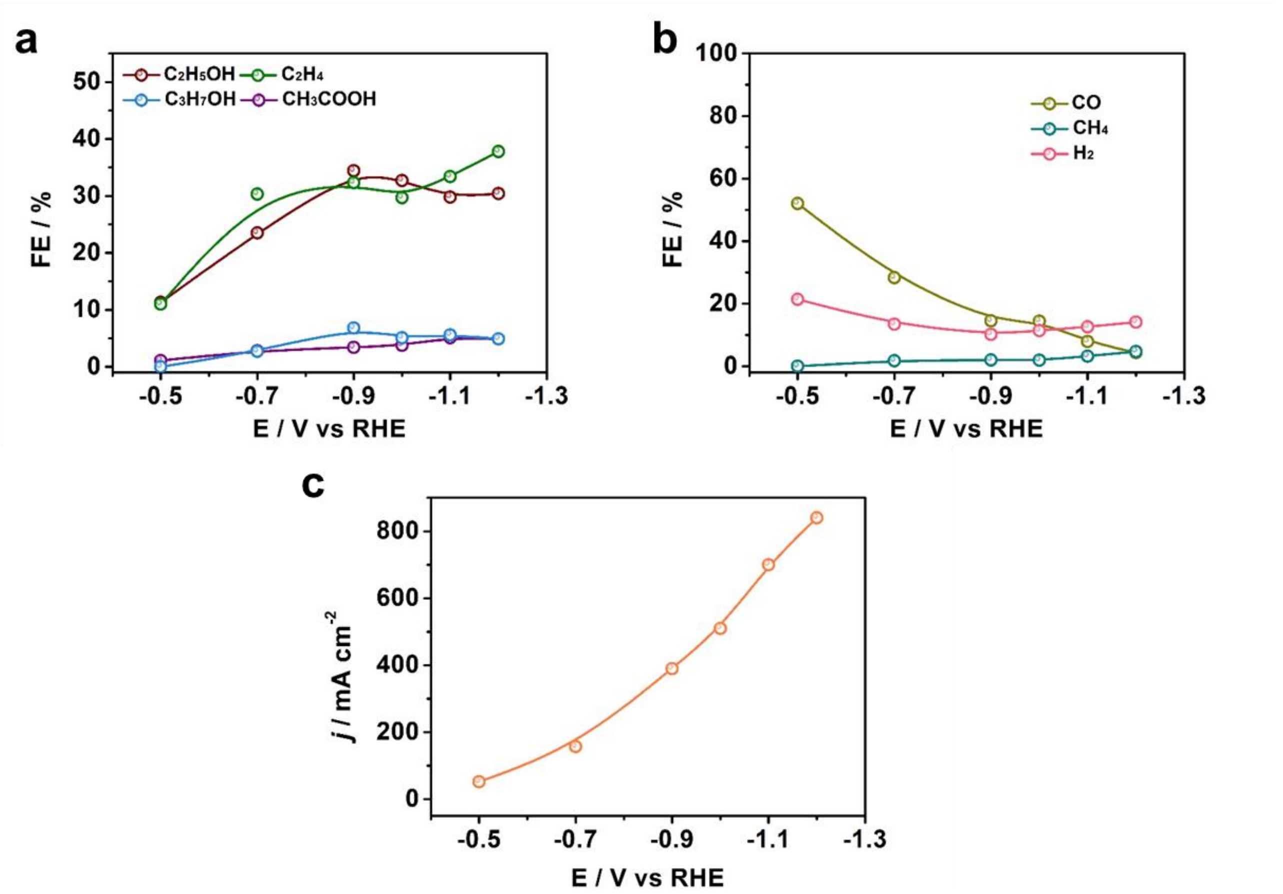

Figure S25. (a) The FE of C2+ products at different potentials over R-8-Cu-4. (b) The FE of H_2 and C1 products at different potentials over R-8-Cu-4. (c) The current density for R-8-Cu-4 at different potentials.

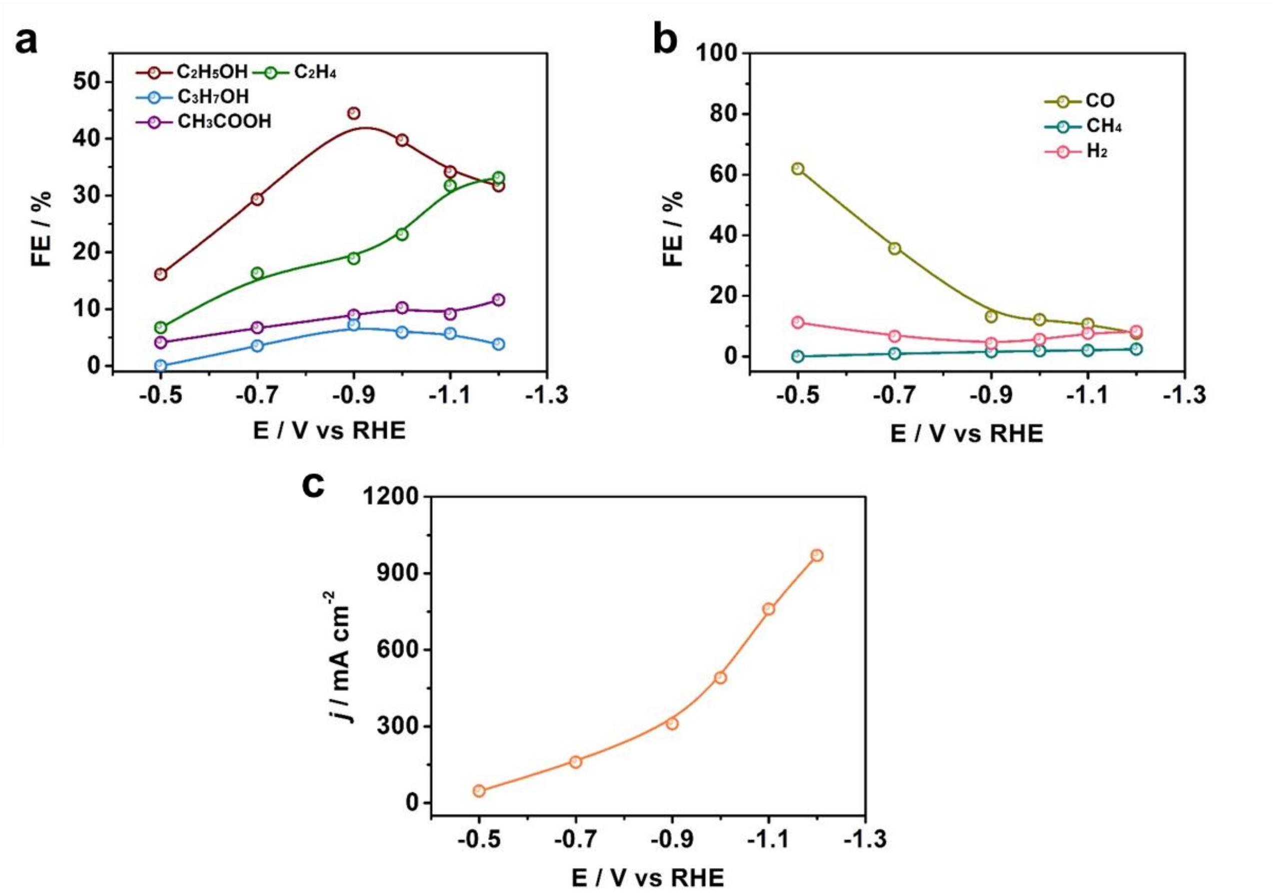

Figure S26. (a) The FE of C2+ products at different potentials over R-8-Cu-16. (b) The FE of H_2 and C1 products at different potentials over R-8-Cu-16. (c) The current density over R-8-Cu-16 at different potentials.

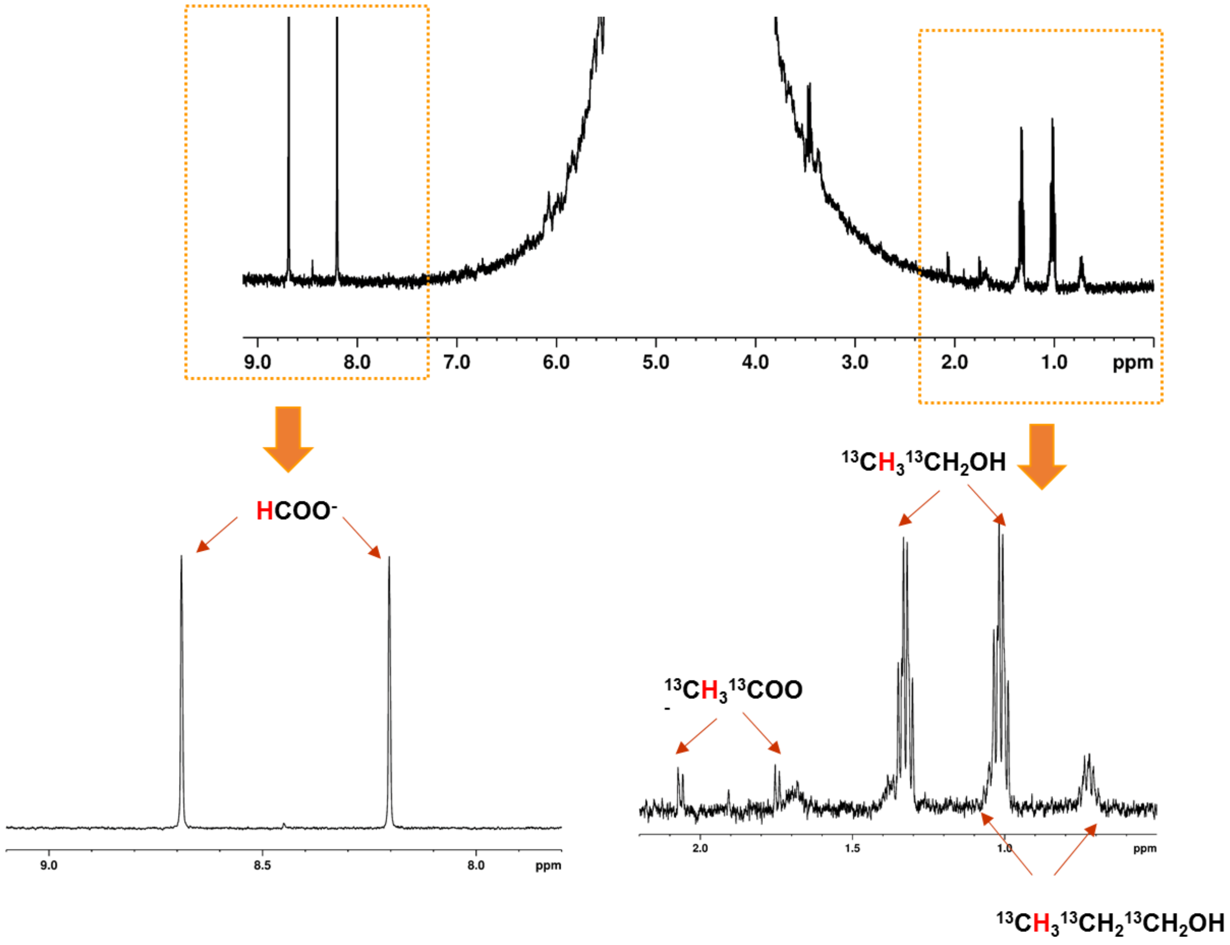

Figure S27. ^{1}H NMR spectra of the liquid products using $^{13}CO_2$ as gas source.

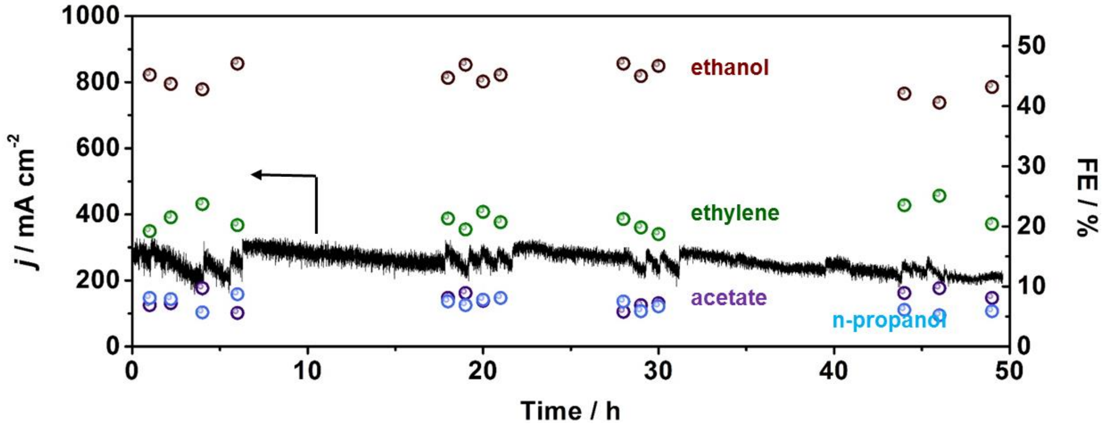

Figure S28. The current density and FE of C2+ oxygenates on R-8-Cu-12 at -0.9 V vs. RHE with 50-hour potentiostatic electrolysis tests. The PTFE membrane was used as the GDL. These fluctuations were caused by the replacement of new electrolyte.

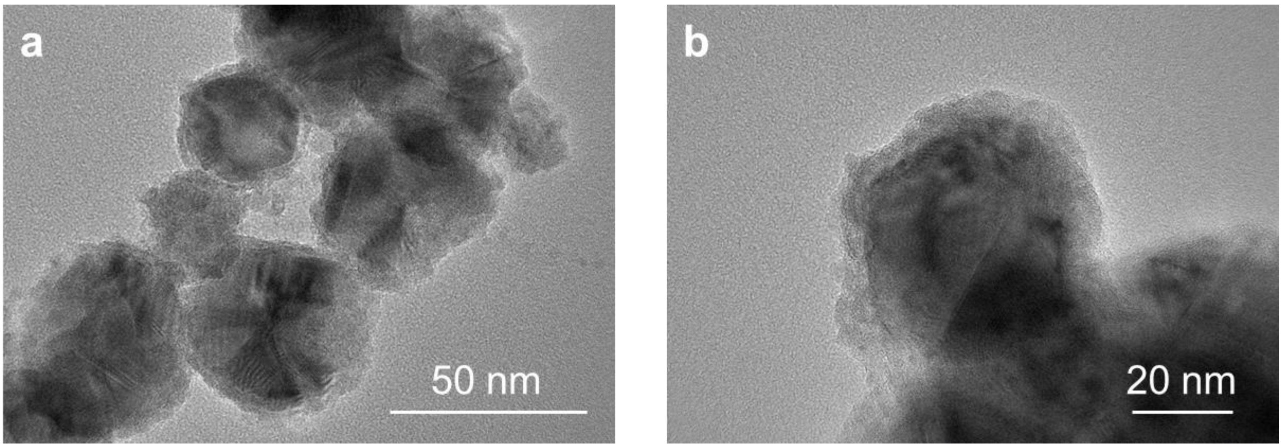

Figure S29. (a, b) The TEM and HR-TEM images of R-8-Cu-12 after CO_2RR.

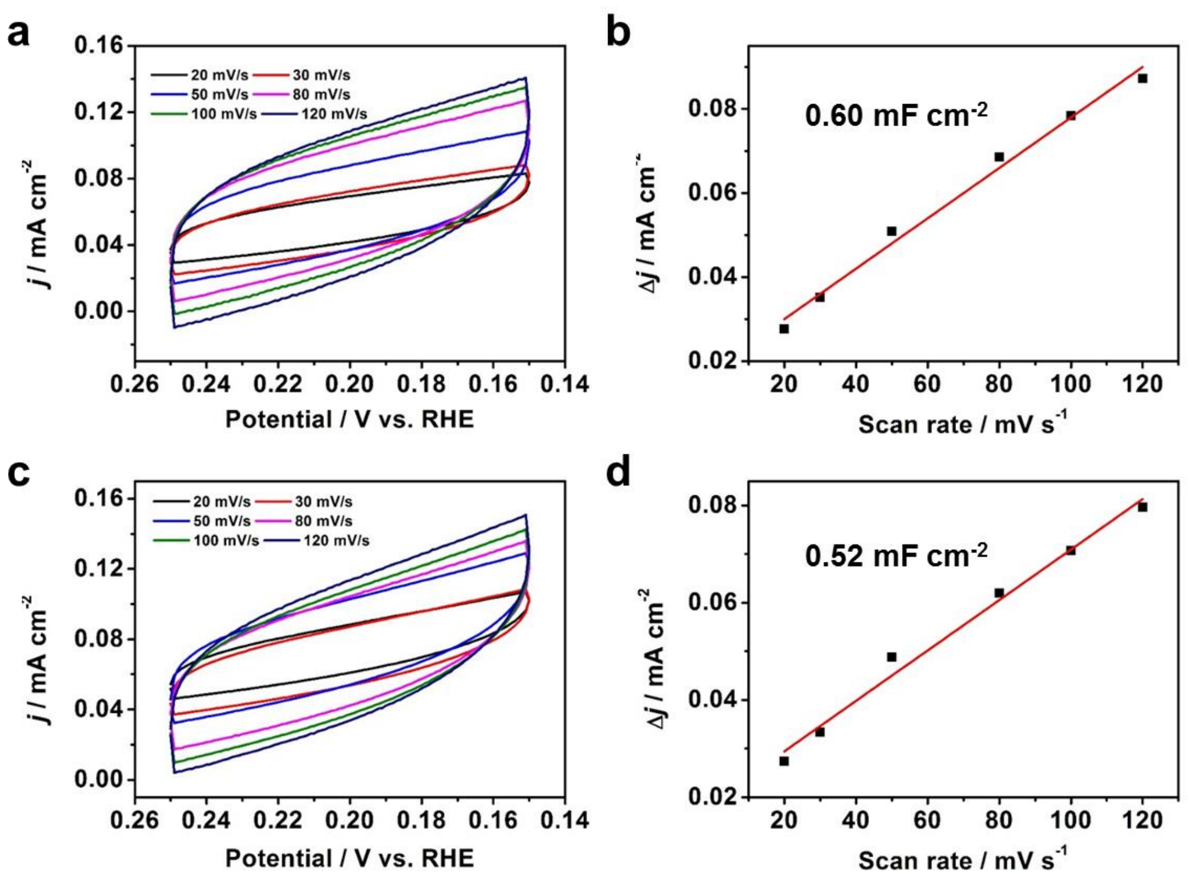

Figure S30. (a) The cyclic voltammetry at different scan rates over R-Cu-np. (b) The charging current density differences plotted against the scan rates over R-Cu-np. (c) The cyclic voltammetry at different scan rates over R-8-Cu-12. (d) The charging current density differences plotted against the scan rates of R-8-Cu-12.

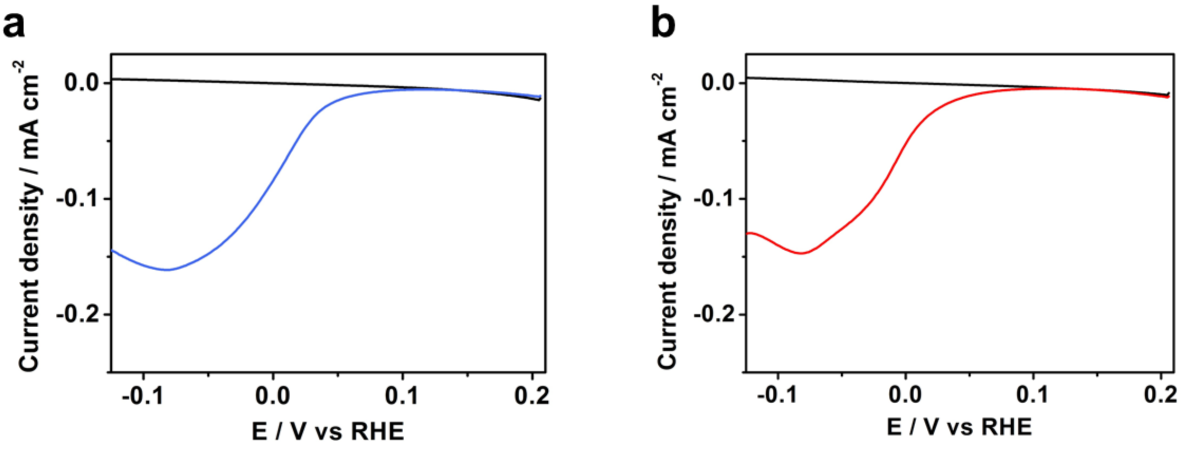

Figure S31. (a) The cyclic voltammetry over R-Cu-np in 100 mM $HClO_4$ + 1 mM $Pb(ClO_4)_2$. (b) The cyclic voltammetry over R-8-Cu-12 in 100 mM $HClO_4$ + 1 mM $Pb(ClO_4)_2$.

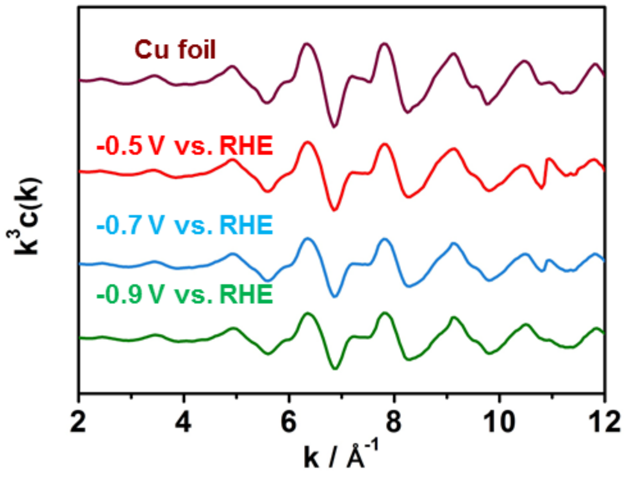

Figure S32. Cu K-edge extended XAFS oscillation function **k**^{3}c(**k**) over R-8-Cu-12 at different potentials.

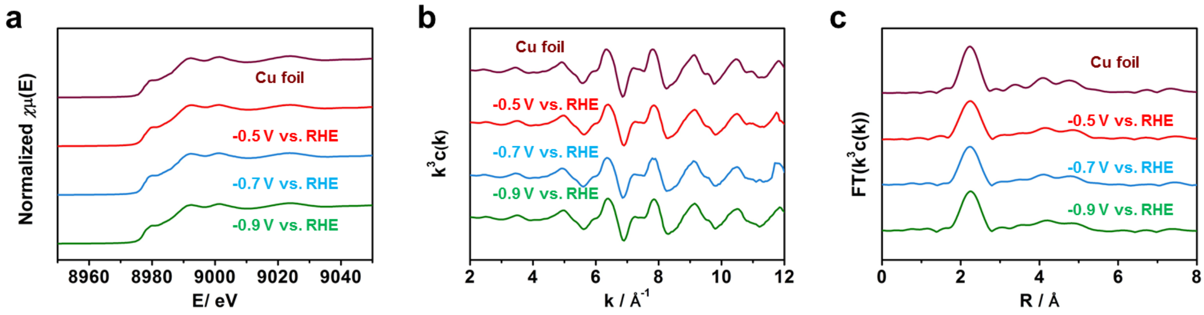

Figure S33. (a) XANES spectra at the Cu K-edge for R-Cu-np at different potentials during CO_2 electrolysis. (b) Cu K-edge extended XAFS oscillation function $\mathbf{k}^3c(\mathbf{k})$ for R-Cu-np at different potentials during CO_2 electrolysis. (c) The corresponding Fourier transforms FT($\mathbf{k}^3c(\mathbf{k})$) for R-Cu-np at different potentials during CO_2 electrolysis.

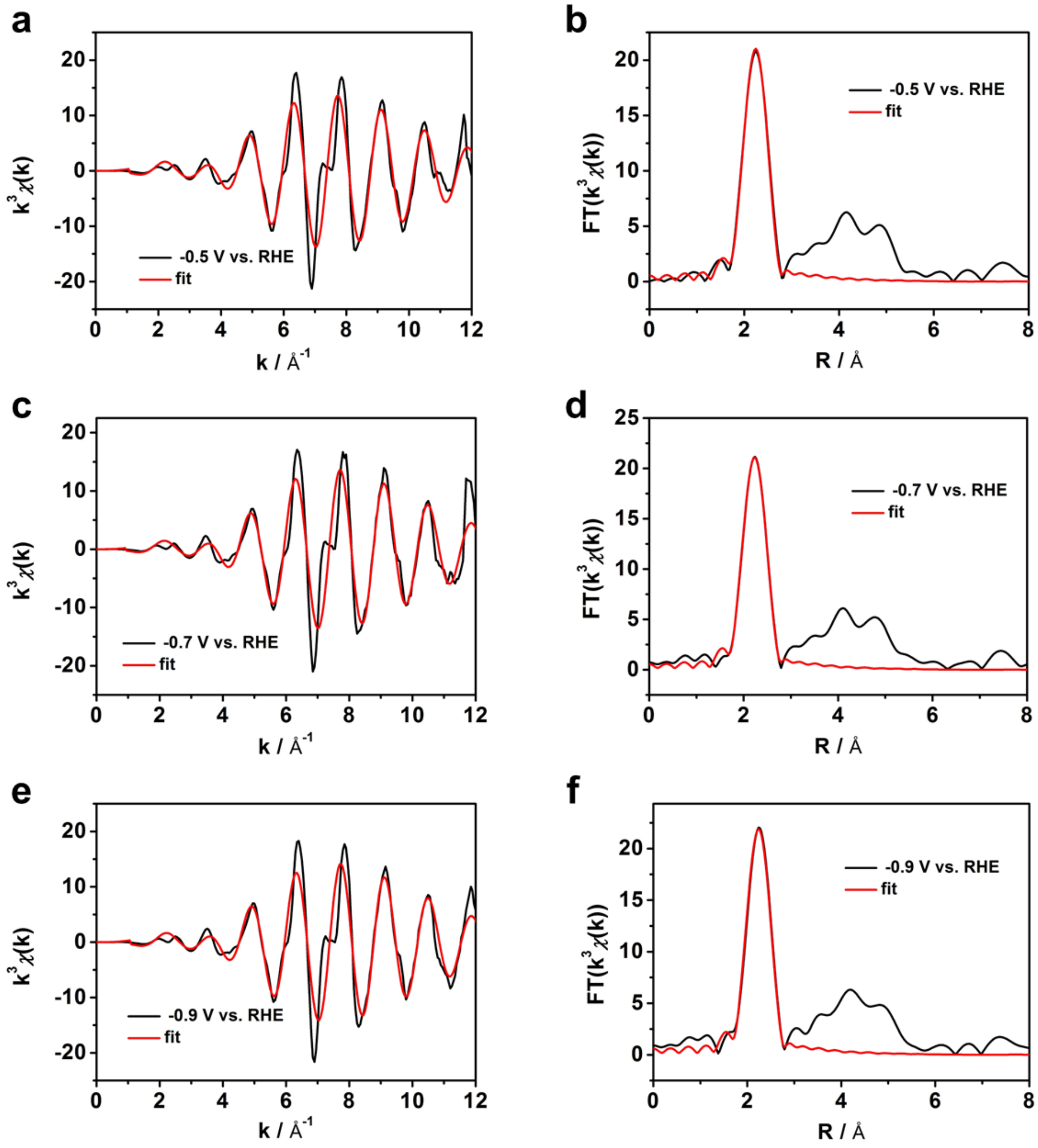

Figure S34. The EXAFS data fitting results of R-Cu-np at different potentials during CO_2 electrolysis.

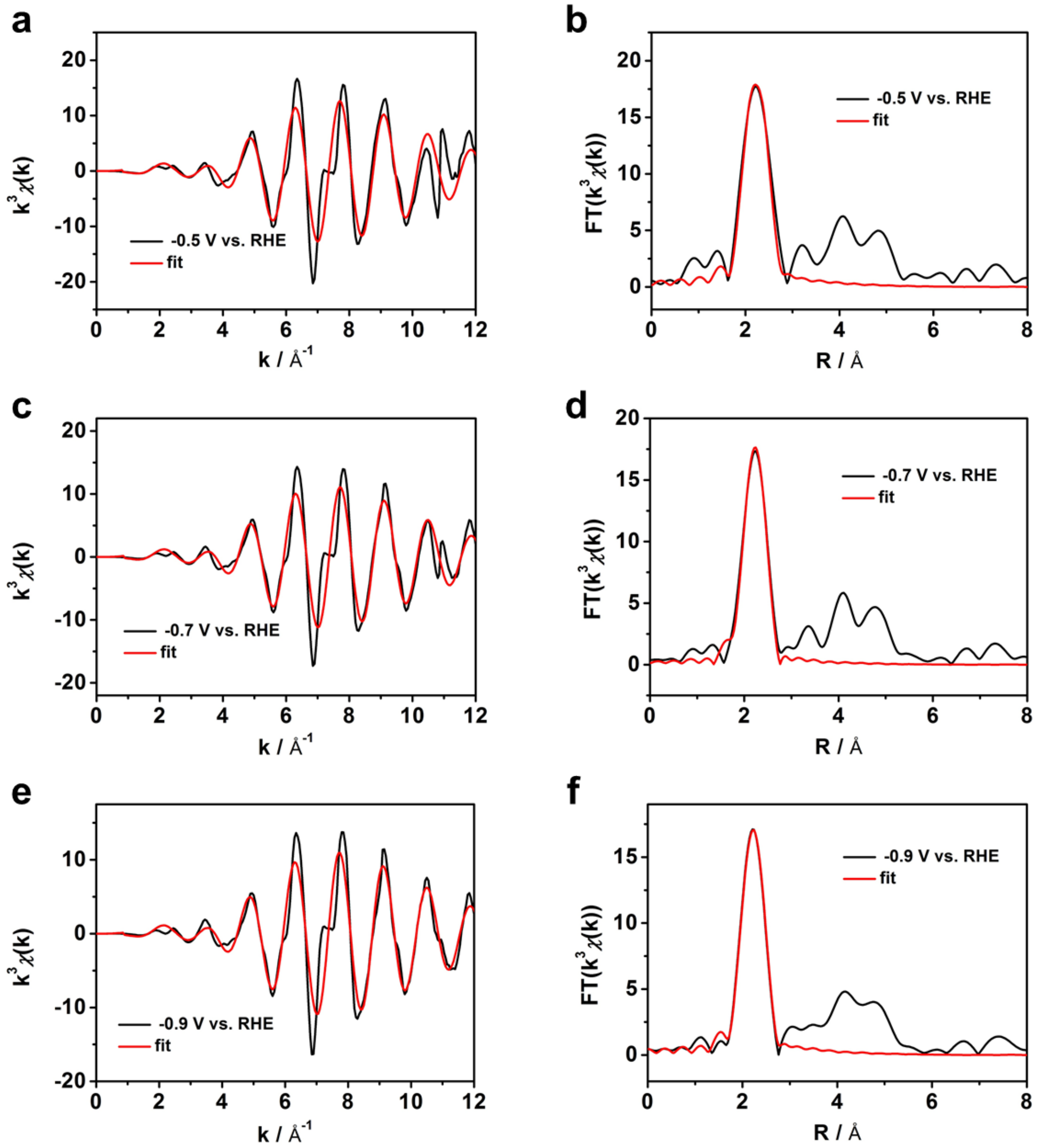

Figure S35. The EXAFS data fitting results of R-8-Cu-12 at different potentials during CO_2 electrolysis.

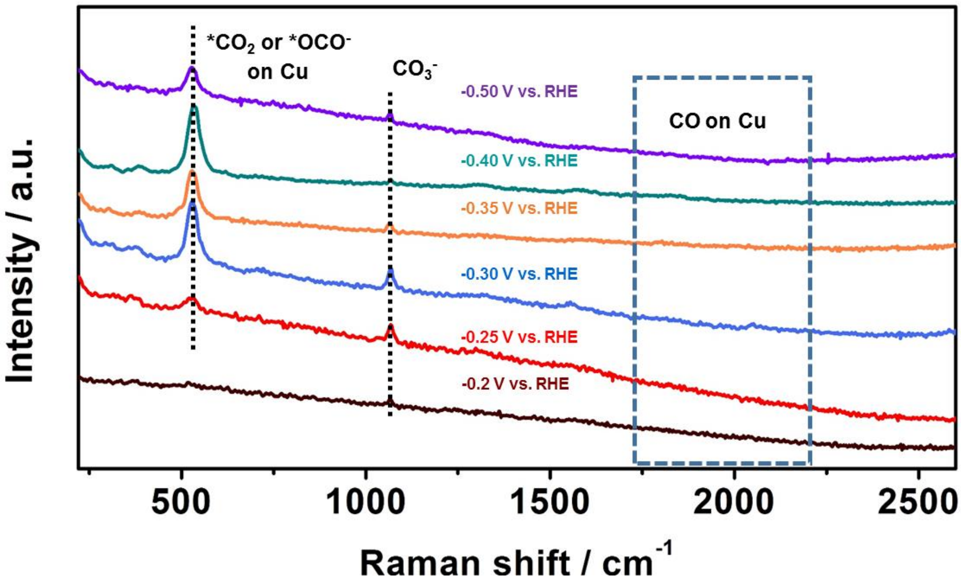

Figure S36. The in-situ SERS for R-Cu-np at different potentials during CO_2 electrolysis.

According to previous reports,[S8, S9] the Raman peaks at about 300~400 cm^{-1} may be related to the *COOH or Cu-CO stretch.

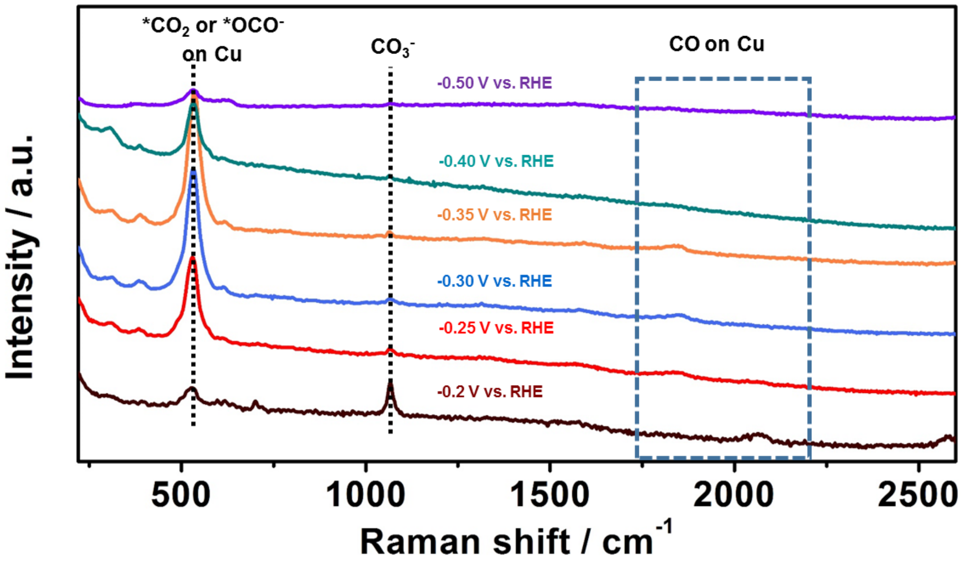

Figure S37. The in-situ SERS for R-8-Cu-12 at different potentials during CO_2 electrolysis.

According to previous reports,[S8, S9] the Raman peaks at about 300~400 cm^{-1} may be related to the *COOH or Cu-CO stretch.

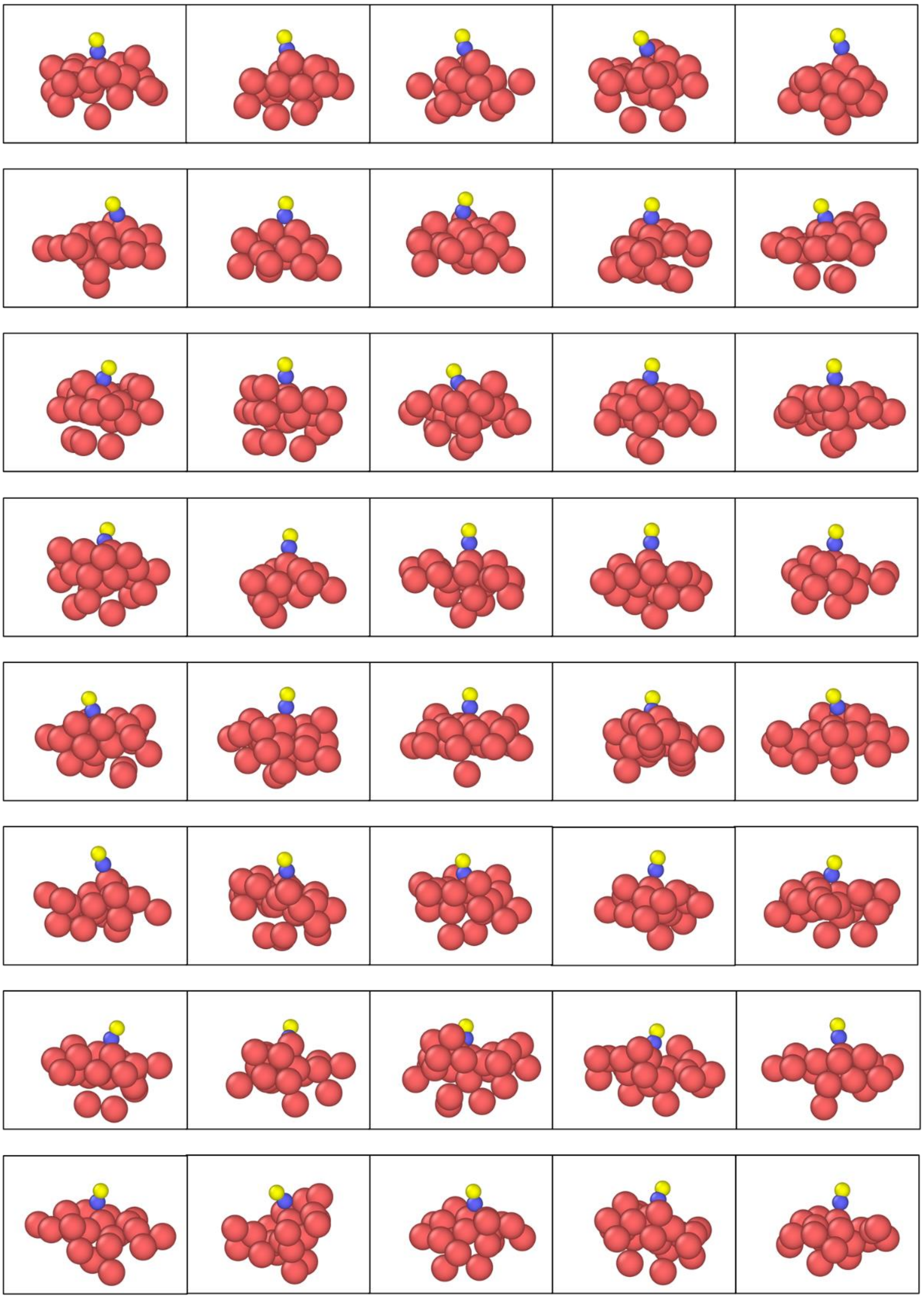

Figure S38. The optimized structures of *CO adsorption on the structures generated from cut models.

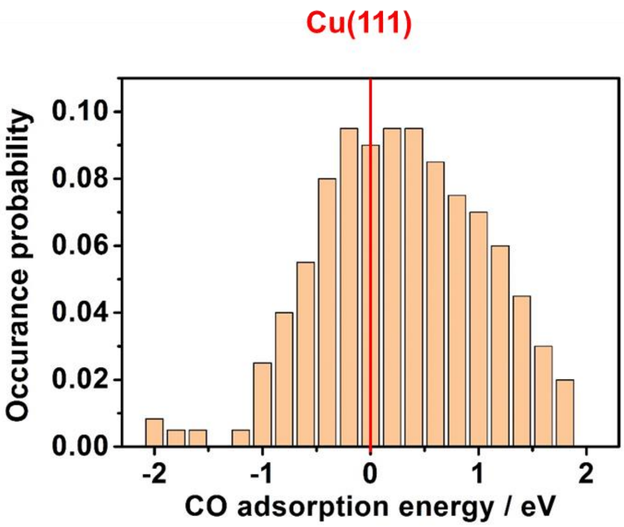

Figure S39. The distribution for CO adsorption energy on amorphous Cu. For the value of energy, we use the relative value to Cu (111).

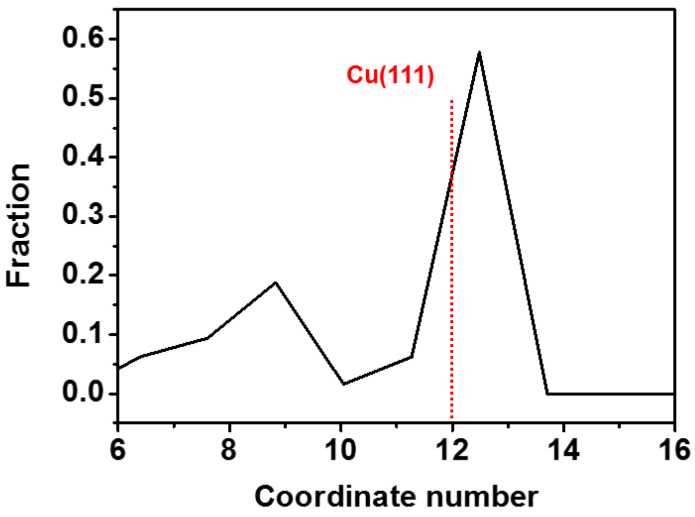

Figure S40. The distribution of CN in amorphous Cu.

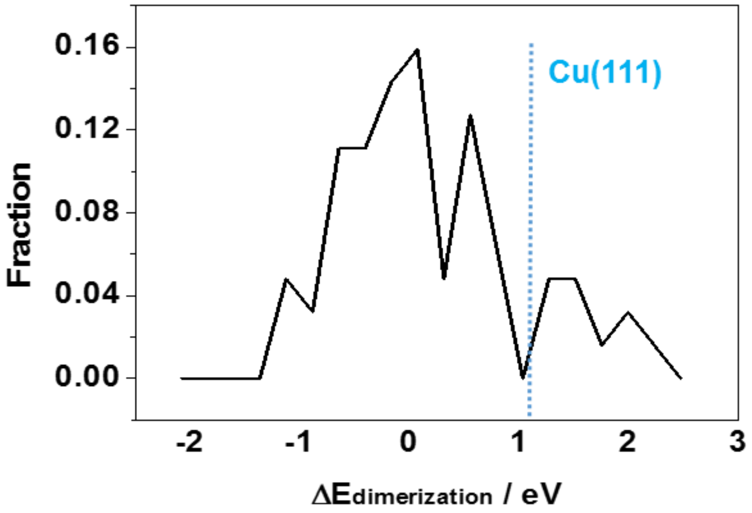

Figure S41. The distribution of energy barrier for dimerization on amorphous Cu.

The energy barriers of the dimerization over the amorphous Cu (64 sites) were studied. We can observe that the energy barriers of dimerization over the most of the sites were lower than that of the Cu (111). Thus, we can conclude that the C-C coupling step can be enhanced over the amorphous Cu.

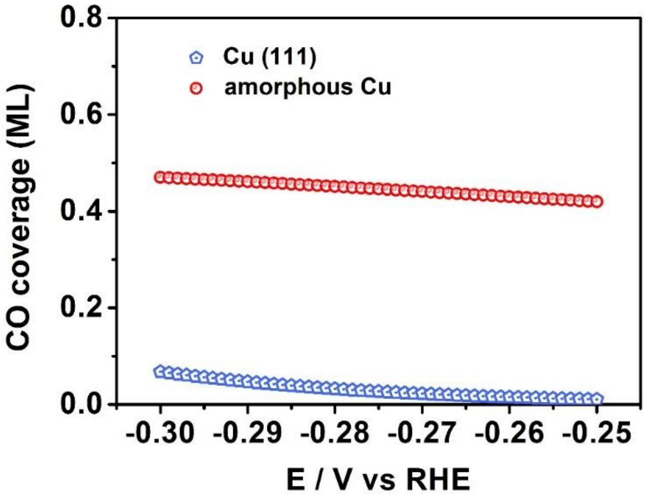

Figure S42. The coverage of CO on Cu (111) and amorphous Cu.

Table S1. Structural parameters of R-Cu-np and R-8-Cu-12 at different potentials extracted from the EXAFS fitting.

Sample	Potential (V vs RHE)	Scattering pair	CN	R(Å)	$\sigma^2 (10^{-3} Å^2)$	ΔE_0(eV)	R
R-Cu-np		Cu-Cu	9.9(3)	2.56(2)	5.0(1)	5.3(3)	0.01
R-Cu-np	-0.5	Cu-Cu	10.1(5)	2.56(2)	5.6(3)	5.3(1)	0.01
R-Cu-np	-0.7	Cu-Cu	9.9(4)	2.56(2)	5.8(6)	5.3(2)	0.01
R-Cu-np	-0.9	Cu-Cu	10.0(2)	2.56(2)	6.1(2)	5.3(4)	0.01
R-8-Cu-12		Cu-Cu	8.6(3)	2.56(2)	5.0(5)	6.0(1)	0.01
R-8-Cu-12	-0.5	Cu-Cu	8.7(3)	2.56(2)	5.5(3)	6.0(3)	0.01
R-8-Cu-12	-0.7	Cu-Cu	8.6(5)	2.56(2)	5.8(4)	6.0(4)	0.01
R-8-Cu-12	-0.9	Cu-Cu	8.7(5)	2.56(2)	6.2(5)	6.0(1)	0.01

S_0^2 is the amplitude reduction factor S_0^2=0.85; CN is the coordination number; R is interatomic distance (the bond length between central atoms and surrounding coordination atoms); σ^2 is Debye-Waller factor (a measure of thermal and static disorder in absorber-scatterer distances); ΔE_0 is edge-energy shift (the difference between the zero kinetic energy value of the sample and that of the theoretical model). R factor is used to value the goodness of the fitting.

Table S2. Comparision of C2+ oxygenate products in CO_2RR on various Cu-based catalysts.

**Samples**	**E vs. RHE**	**FE of C2+ oxygenates(%)**	**J of C2+ oxygenates(%) (mA cm^{-2})**	**references**
R-8-Cu-12	-0.9	65.3	209.2	This work
Ce(OH)x/Cu	-0.7	46.5	139.5	S10
FeTPP[Cl]/Cu	-0.82	45	135	S11
Cu-F	-0.89	12	192	S12
NGQ/Cu-nr	-0.9	54.4	152	S13
N-C/Cu	-0.8	56	168	S9
CSVE-Cu	-0.92	34.6	138.4	S14
Cu-Ag	-0.58	25	75	S15
Nanoporous Cu	-0.67	16.6	108	S16

References

S1 Behler, J. First Principles Neural Network Potentials for Reactive Simulations of Large Molecular and Condensed Systems. *Angew. Chem. Int. Ed.* **56**, 12828-12840 (2017).

S2 Behler, J., Parrinello, M. Generalized neural-network representation of high-dimensional potential-energy surfaces. *Phys. Rev. Lett.* **98**, 146401 (2007).

S3 Wang, H., Zhang, L., Han, J., & Weinan, E. DeePMD-kit: a deep learning package for many-body potential energy representation and molecular dynamics. *Computer Phys. Commun* **228**, 178-184 (2018).

S4 Giannozzi, P. *et al.*, QUANTUM ESPRESSO: a modular and open-source software project for quantum simulations of materials. *J. Phys. Condens. Matter.* **21**, 395502 (2009).

S5 Plimpton, S. Fast Parallel Algorithms for Short-Range Molecular Dynamics. *J. Comput. Phys* **117**, 1-19 (1995).

S6 Nørskov, J. K. R. *et al.*, Origin of the Overpotential for Oxygen Reduction at a Fuel-Cell Cathode. *J. Phys. Chem. B* **2004**, 17886-17892 (2004).

S7 Peterson, A. A., Abild-Pedersen, F., Studt, F., Rossmeisl, J., Nørskov, J. K. How copper catalyzes the electroreduction of carbon dioxide into hydrocarbon fuels. *Energy Environ. Sci.* **3**, 1311-1315 (2010).

S8 Shan, W. *et al.*, In Situ Surface-Enhanced Raman Spectroscopic Evidence on the Origin of Selectivity in CO_2 Electrocatalytic Reduction. *ACS Nano*, **14**, 11363-11372 (2020).

S9 Wang, X. *et al.*, Efficient electrically powered CO_2-to-ethanol via suppression of deoxygenation. *Nature Energy*, **5**, 478-486 (2020).

S10 Luo, M. *et al.*, Hydroxide promotes carbon dioxide electroreduction to ethanol on copper via tuning of adsorbed hydrogen. *Nat. Commun.* **10**, 5814 (2019).

S11 Li, F. *et al.*, Cooperative CO_2-to-ethanol conversion via enriched intermediates at molecule-metal catalyst interfaces. *Nat. Catal.* **3**, 75-82 (2020).

S12 Ma, W. *et al.*, Electrocatalytic reduction of CO_2 to ethylene and ethanol through hydrogen-assisted C-C coupling over fluorine-modified copper. *Nat. Catal.* **3**, 478-487 (2020).

S13 Chen, C. *et al.*, Highly Efficient Electroreduction of CO_2 to C2+ Alcohols on Heterogeneous Dual Active Sites. *Angew. Chem. Int. Ed.* **59**, 16459-16464 (2020).

S14 Zhuang, T.-T. *et al.* Steering post-C–C coupling selectivity enables high efficiency electroreduction of carbon dioxide to multi-carbon alcohols. *Nat. Catal.* **1**, 421-428 (2018).

S15 Hoang, T. T. H. *et al.*, Nanoporous Copper-Silver Alloys by Additive-Controlled Electrodeposition for the Selective Electroreduction of CO_2 to Ethylene and Ethanol. *J. Am. Chem. Soc.* **140**, 5791-5797 (2018).

S16 Lv, J. J. *et al.*, A Highly porous copper electrocatalyst for carbon dioxide reduction. *Adv. Mater.* **30**, e1803111 (2018).
