# Supplementary material for: Oxidation of metallic Cu by supercritical CO2 and control synthesis of amorphous nano-metal catalysts for CO2 electroreduction
Source: Nat Commun. 2023 Feb 25;14:1092. doi: 10.1038/s41467-023-36721-8 (PMC9968285; doi:10.1038/s41467-023-36721-8)
Supplement: Supplementary file 2 — Description of Additional Supplementary Files [file 41467_2023_36721_MOESM2_ESM.pdf]

Supplementary Movie 1. Simulation of disordered formation of Cu-np under SC CO_2.
Supplementary Movie 2. Simulation of surface oxidation and O atomic penetration on the Cu-np under SC CO_2.
Supplementary Movie 3. Simulation of surface oxidation on the Cu-np under SC CO_2.
Supplementary Movie 4. Simulation of O atomic penetration on the Cu-np under SC CO_2.
Supplementary Movie 5. Simulation of O departing on the amorphous Cu_xO.
Supplementary Movie 6. The 40 random positions on the amorphous Cu model.
